# Phylogenetic and morphological studies in *Xylodon* (Hymenochaetales, Basidiomycota) with the addition of four new species

**DOI:** 10.3897/mycokeys.47.31130

**Published:** 2019-02-28

**Authors:** Janett Riebesehl, Eugene Yurchenko, Karen K. Nakasone, Ewald Langer

**Affiliations:** 1 Department of Ecology, University of Kassel, Heinrich-Plett-Str. 40, DE-34132, Kassel, Germany University of Kassel Kassel Germany; 2 Department of Biotechnology, Polessky State University, Dnyaprouskai flatylii str. 23, BY-225710, Pinsk, Belarus Polessky State University Pinsk Belarus; 3 Center for Forest Mycology Research, Northern Research Station, U.S. Forest Service, One Gifford Pinchot Drive, Madison, WI 53726-2398, USA U.S. Forest Service Madison United States of America

**Keywords:** Agaricomycetes, corticioid fungi, Schizoporaceae, *
Schizopora
*, *
Odontia ambigua
*, *
Xylodon echinatus
*

## Abstract

*Xylodon* (Hymenochaetales, Basidiomycota) is the largest segregate genus of *Hyphodontia* s.l. Based on molecular and morphological data, 77 species are accepted in *Xylodon* to date. Phylogenetic analyses of ITS and 28S sequences, including 38 new ITS and 20 28S sequences of *Xylodon* species, revealed four species new to science. The new taxa *X.exilis*, *X.filicinus*, *X.follis* and *X.pseudolanatus* from Taiwan, Nepal, Réunion, Belize, and USA are described and illustrated. In addition, species concepts for *Odontiavesiculosa* from New Zealand and *Xylodonlanatus* from U.S.A. are revised and the new name *X.vesiculosus* is proposed. Phylogenetic analyses of the ITS region placed *X.spathulatus*, *X.bubalinus* and *X.chinensis* in a strongly supported clade and demonstrated that they are conspecific. *Palifer* and *Odontiopsis* are synonymised under *Xylodon* based on morphological and sequence data. The following new combinations are proposed: *X.erikssonii*, *X.gamundiae*, *X.hjortstamii*, *X.hyphodontinus*, *X.septocystidiatus* and *X.verecundus*. Line drawings of *X.cystidiatus*, *X.hyphodontinus*, *X.lanatus* and *X.vesiculosus*, as well as photographs of *X.raduloides* basidiomata, are provided. A key to *X.lanatus* and similar species is presented.

## Introduction

The corticioid fungal genus *Xylodon* (Pers.) Gray, based on the generic type *X.quercinus* (Pers.) Gray, was described in 1801 by Persoon as Sistotremasect.Xylodon and belongs in the Hymenochaetales (Basidiomycota). Species of *Xylodon* were usually placed in *Hyphodontia* J. Erikss. until Hjortstam and Ryvarden (2002, 2009) reorganised *Hyphodontia* s.l. into different genera based on morphological features.

The most recent generic description of *Xylodon* was published by Riebesehl and Langer (2017). With few exceptions, the hymenophore in *Xylodon* is odontioid or poroid with many different cystidia types and basidiospore shapes.

*Xylodon* spp. are primarily wood decomposers, causing a white-rot of angiosperms and gymnosperms (Eriksson and Ryvarden 1976, Yurchenko and Wu 2014). A few species have been collected on brown-rotten spruce stumps, palms or palm tree inflorescences, bamboo, ferns and on the herbaceous *Staehelinadubia* L. and *Fallopiasachalinensis* (F.Schmidt) Ronse Decr. (Burdsall and Nakasone 1981, Langer 1994, Nordén et al. 1999, Kotiranta and Saarenoksa 2000, Boidin and Gilles 2003, Hjortstam et al. 2005, Xiong et al. 2010, Jo et al. 2018). *Xylodon* has a worldwide distribution, with both cosmopolitan species and species restricted to a limited geographic area.

*Palifer* Stalpers & P.K.Buchanan (1991), based on *Peniophoraverecunda* G.Cunn. from New Zealand, is another segregate genus of *Hyphodontia* s.l. recognised by Hjortstam and Ryvarden (2009). It is characterised by encrusted cystidia and remained monotypic until 2007 when three species were transferred to the genus (Hjortstam and Ryvarden 2007a). After a thorough morphological study of *Palifer* species and related taxa, Gorjón (2012) concluded that *Palifer* was probably a synonym of *Xylodon* but did not propose any new combinations. *Palifer* is represented by only one nuclear ribosomal internal transcribed spacer (ITS) sequence in the public record and phylogenetic analyses showed it to be embedded in *Xylodon* (Larsson et al. 2006). Riebesehl and Langer (2017), however, declined to synonymise *Palifer* with *Xylodon* based on one DNA sequence alone and chose to emphasise its morphological features.

*Odontiopsis*Hjortstam and Ryvarden (1980) is based on the type species *O.hyphodontina* Hjortstam & Ryvarden from Tanzania. It is characterised by an odontioid hymenium, encrusted hyphae projecting from the aculei, stout basidia and globose to subglobose basidiospores. Hjortstam and Ryvarden (1980) mentioned hyphal and basidial similarities with *Schizopora* and *Hyphodontia*.

In this study, we conducted an in-depth phylogenetic study of 36 *Xylodon* species represented by 96 strains or collections, including 58 new ITS and large subunit (28S) ribosomal DNA sequences. Phylogenetic analyses of the ITS and 28S sequence data uncovered four new taxa, *X.exilis*, *X.filicinus*, *X.follis* and *X.pseudolanatus*, that are described and illustrated. In addition, the species complex of *X.spathulatus* was identified and resulted in the synonymisation of two taxa. The genera *Palifer* and *Odontiopsis* are re-evaluated and placed in synonymy with *Xylodon*, resulting in a number of new combinations. Morphological studies in *Xylodonlanatus* and *Odontiavesiculosa* were conducted and a key to morphologically similar species is provided. Line drawings of *X.cystidiatus*, *X.hyphodontinus* and *X.vesiculosus* are presented and *X.vesiculosus* is described.

## Methods

### Molecular study

Pieces of dried basidiomata served as material for DNA extractions with the E.Z.N.A.® Fungal DNA Mini Kit (Omega Bio-Tek, VWR, USA). Two nuclear ribosomal DNA markers were used in this study: the ITS region and the D1-D2 domains of 28S. The ITS region includes the internal transcribed spacers 1 and 2 as well as the intercalary 5.8S rRNA gene. For amplification of ITS, different combinations of the following primers were used: ITS1-F (Gardes and Bruns 1993), ITS1, ITS2, ITS3, ITS4, ITS5 (White et al. 1990) and ALR0 (Collopy et al. 2001). The last one was modified in one position (Riebesehl and Langer 2017). NL1, NL4 (O’Donnell 1993), LR0R (Bunyard et al. 1996) and LR5 (Vilgalys and Hester 1990) were used, also in different combinations, for the amplifications of the D1-D2 domains of 28S. PCR products were purified with innuPREP PCRpure Kit (Analytik Jena, Berlin, Germany) and the DNA sequencing was implemented by Eurofins Genomics (Ebersberg, Germany).

Newly generated sequences were edited with MEGA7 (Kumar et al. 2016). Their quality was checked following the five guidelines by Nilsson et al. (2012) and they were deposited in NCBI GenBank (Benson et al. 2018; Tab. 1). Other sequences used in this study were downloaded from the same database. *Phellinusgabonensis* Decock & Yombiyeni (Hymenochaetales) was chosen as the outgroup for rooting the phylograms. The two different alignments were calculated with MAFFT v.7 (Katoh and Standley 2013), using the L-INS-i algorithm for ITS and G-INS-i for 28S. Minimum Evolution (ME) and Bayesian inference (BI) trees were calculated for both datasets. The ME phylograms were computed with MEGA7, using the Tamura-Nei model (Tamura and Nei 1993) including 1000 bootstrap (BS) replications, partial deletion of gapped positions with 95% site coverage cut-off and other default settings. The BI phylograms were constructed with MrBayes 3.2.6 (Ronquist and Huelsenbeck 2003), using DNA substitution models estimated by MrModeltest 2.4 (Nylander 2004) with 10 million generations and one tree saved for every 1000 generations; other parameters used default settings. A partitioned analysis was done for the ITS alignment with independent DNA substitution models and parameter values for ITS1, 5.8S and ITS2. MEGA7 and FigTree 1.4.2 (Rambaut 2012) were used for processing the phylograms.

### Morphological study

The studied specimens are deposited in herbaria CFMR, FR, KAS, LIP, MSK, and TUB (acronyms follow Index Herbariorum, http://sweetgum.nybg.org/science/ih). Morphological descriptions and figures employed dried basidiomata. Preparations in 3% potassium hydroxide (KOH) aqueous solution were used for microscopic measurements and most drawings. Crystalline deposits on hyphae were additionally examined in Melzer’s reagent (Mz) and tap water. Amyloid and dextrinoid reactions of basidiospores were tested with Mz. Spore wall cyanophily was determined in Cotton Blue-Lactophenol solution (CBL). The following abbreviations are used to describe arithmetic averages for 30 basidiospores, randomly selected in squash preparations of one specimen: L – spore length, W – spore width, Q = length/width ratio.

## Results

### Phylogeny

**Figure 1.**
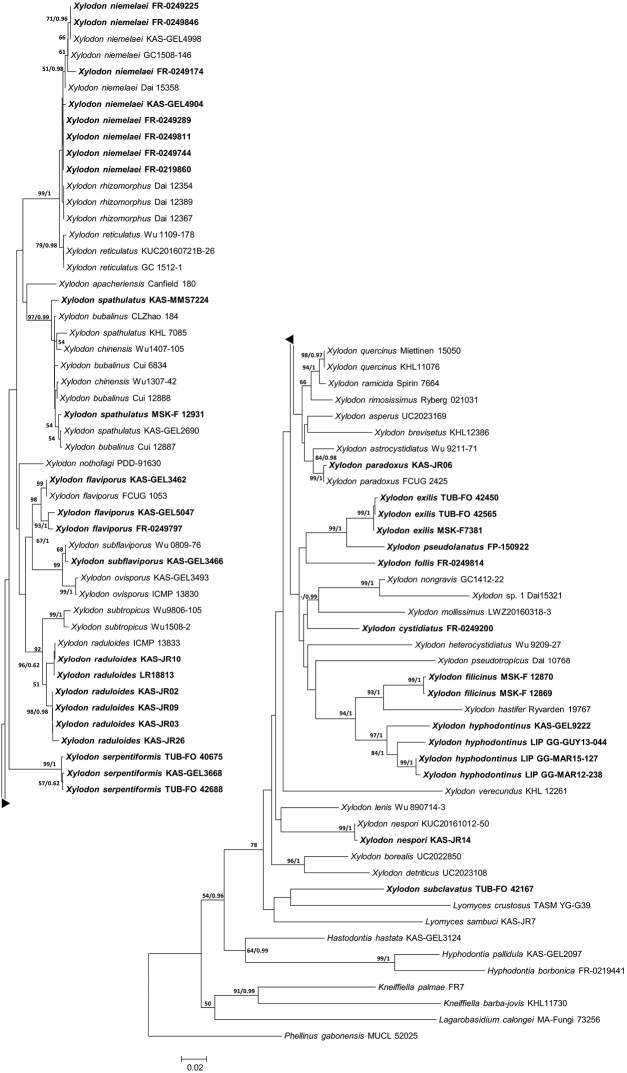
ITS-based Minimum Evolution phylogram for *Xylodon* and allied species. Bootstrap values >50 are shown next to the branches. The second number, if present, represents posterior probabilities received from BI analysis. Scale bar indicates estimated number of substitutions per site. Sequences generated in this study are shown in bold. Voucher numbers and species names are indicated in Table 1.

**Figure 2.**
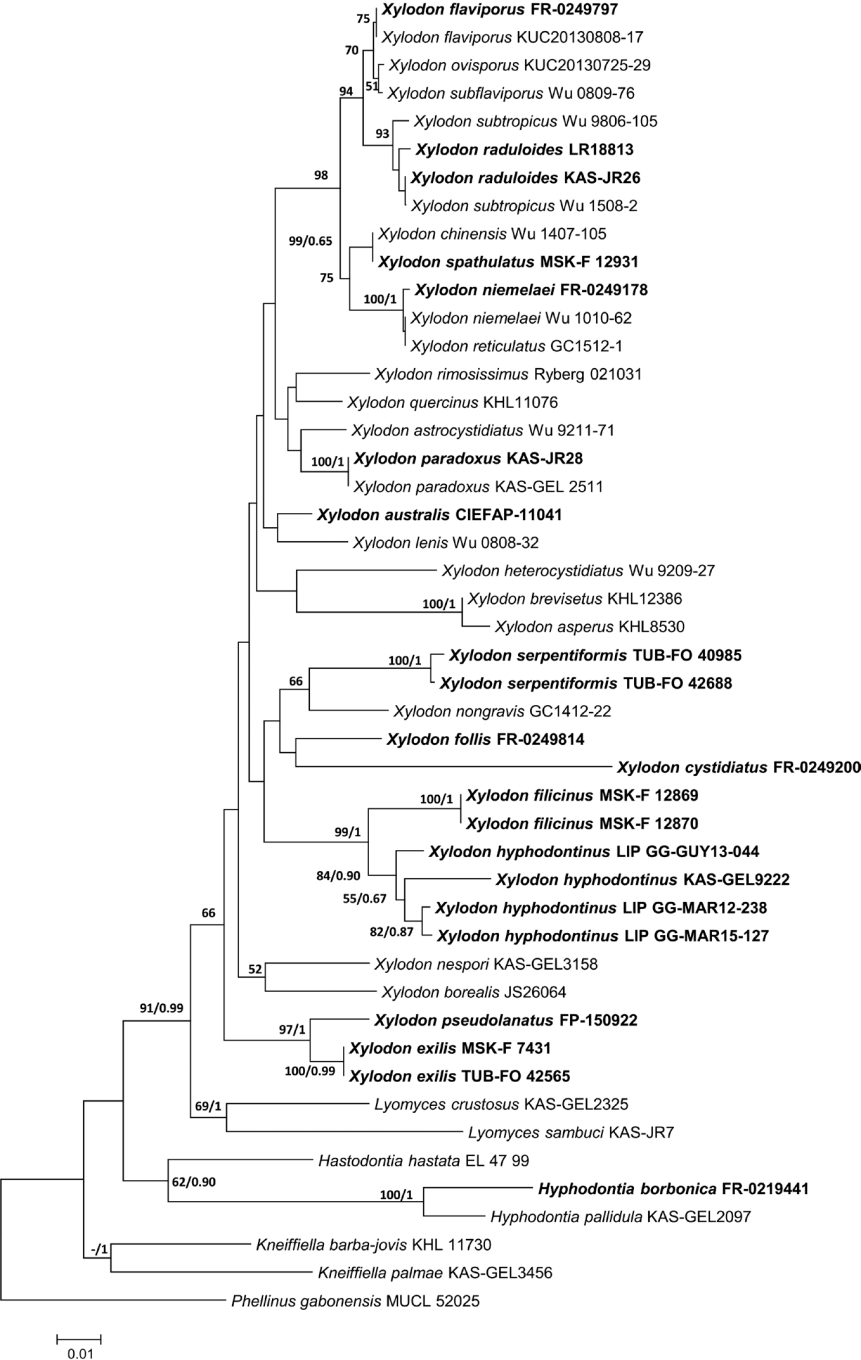
28S-based Minimum Evolution phylogram for *Xylodon* and allied species. Bootstrap values >50 are shown next to the branches. The second number, if present, represents posterior probabilities received from BI analysis. Scale bar indicates estimated number of substitutions per site. Sequences generated in this study are shown in bold. Voucher numbers and species names are indicated in Table 1.

The aligned ITS data matrix consisted of 92 taxa and 847 positions. The partial deletion of gapped positions resulted in 463 positions that were used in the ME phylogenetic analysis. The data matrix was partitioned as follows: ITS1 = positions 1–373, 5.8S rRNA gene = 374–541 and ITS2 = 542–847. The GTR + G model was used as DNA substitution model for ITS1 and ITS2 and SYM + I for 5.8S in the BI analysis. The aligned data matrix of the D1-D2 domains of 28S rRNA gene consisted of 47 taxa and 634 positions; 532 positions were used in the ME analysis. The GTR + I + G model was chosen as the DNA substitution model for the BI analysis. A high degree of agreement was observed between the ME and BI trees; therefore, the ME phylogram with BS and integrated posterior probability (PP) values from the BI phylogram are presented in Figures 1, 2. The sum of branch lengths in the resulting ME phylograms was 3017 for ITS (Fig. 1) and 1009 for 28S (Fig. 2). Multiple sequence alignments and trees are deposited in TreeBASE (http://purl.org/phylo/treebase/phylows/study/TB2:S23512).

The ITS phylogram (Fig. 1) shows a number of clades with low BS support which is well-documented for *Xylodon* (see Chen et al. 2018, Riebesehl and Langer 2017). Figure 1 includes 83 sequences of *Xylodon* specimens or strains of which 38 were generated in this study. No significant distances were observed amongst sequences of *X.niemelaei*, *X.rhizomorphus*, and *X.reticulatus* nor amongst sequences of *X.spathulatus*, *X.chinensis*, and *X.bubalinus*. The strong BS (99 and 97) and PP (1) values of these two clades indicate that the taxa within each clade may be conspecific. Seven collections of *X.raduloides* form two distinct subclades (96 and 98 BS). Similarly, four collections of *X.flaviporus* formed two subclades (99 and 93 BS). The newly generated ITS sequences show that *X.cystidiatus*, *X.hyphodontinus*, *X.serpentiformis* and *X.subclavatus* form distinct lineages in *Xylodon*. The four new species introduced herein form distinct lineages as well. *Xylodonpseudolanatus* and *X.exilis* are sister groups with 5.9% differences between their ITS sequences (*X.pseudolanatus*: FP-150922 and *X.exilis*: TUB-FO 42565). They cluster in a well-supported clade (99 BS), within a weakly supported lineage that includes *X.follis*. The closest relative of *X.filicinus* is *X.hastifer*; they form a clade (93 BS) that is sister to *X.hyphodontinus* s.l.

The 28S phylogram (Fig. 2) includes 39 sequences of *Xylodon* of which 20 were generated here. Notable new 28S sequences include *X.australis*, *X.hyphodontinus*, *X.serpentiformis*, the four new species described herein, and furthermore *Hyphodontiaborbonica*. As the 28S phylogram features several lineages with low BS support, the clades between the 28S and ITS trees are not identical throughout. Although clearly resolved with ITS sequences, the 28S gene analyses were not able to resolve the closely related *X.raduloides* and *X.subtropicus*. Some clades that were well supported with ITS sequences were also well supported in the 28S phylogram, for example, the *X.niemelaei* and *X.reticulatus* (100 BS) and the *X.chinensis* and *X.spathulatus* (99 BS) clades.

**Table 1.** List of accepted species in *Xylodon* with some closely related species from other genera, including specimens used in the phylogenetic study. Newly generated sequences are shown in bold. *Xylodon* species without available ITS or 28S sequences are marked with ‘not available’ (n.a.); these have to date not been studied using ribosomal sequence data.

Species	Specimen voucher	GenBank accession number		Reference	Country
		ITS	28S		
*Hastodontiahastata* (Litsch.) Hjortstam & Ryvarden	KAS-GEL3124	DQ340311	–	unpublished	Sweden
	EL47/99 (GB)	–	DQ873620	Larsson et al. 2006	Sweden
***Hyphodontiaborbonica* Riebesehl, Langer & Barniske**	**FR-0219441, Holotype**	KR349240	–	Riebesehl et al. 2015	**Réunion**
	–	–	**MH884915**	**This study**	–
*H.pallidula* (Bres.) J.Erikss.	KAS-GEL2097	DQ340317	DQ340372	unpublished	Germany
*Kneiffiellabarba-jovis* (Bull.) P.Karst.	KHL 11730 (GB)	DQ873609	DQ873610	Larsson et al. 2006	Sweden
*K.palmae* Rick ex Hjortstam & Ryvarden	FR7	KP689185	–	Wang et al. 2016	China
	KAS-GEL3456	–	DQ340369	unpublished	Taiwan
*Lagarobasidiumcalongei* M.Dueñas, Tellería, Melo & M.P.Martín	MA-Fungi 73256	NR119737	n.a.	Dueñas et al. 2009	Azore Islands
*Lyomycescrustosus* (Pers.) P.Karst.	TASM YG-G39	MF382993	–	Gafforov et al. 2017	Uzbekistan
	KAS-GEL2325	–	DQ340354	unpublished	Germany
*L.sambuci* (Pers.) P.Karst.	KAS-JR7	KY800402	KY795966	Yurchenko et al. 2017	Germany
*Phellinusgabonensis* Decock & Yombiyeni	MUCL 52025	HM635715	HM635690	Yombiyeni et al. 2011	Gabon
*Xylodonadhaerisporus* (Langer) Hjortstam & Ryvarden	–	n.a.	n.a.	–	–
*X.anmashanensis* (Yurchenko, H.X.Xiong & Sheng H.Wu) Riebesehl, Yurchenko & Langer	–	n.a.	n.a.	–	–
*X.apacheriensis* (Gilb. & Canf.) Hjortstam & Ryvarden	Canfield 180, Holotype	KY081800	n.a.	Riebesehl and Langer 2017	USA, Arizona
*X.archeri* (Berk.) Kuntze	–	n.a.	n.a.	–	–
*X.asperus* (Fr.) Hjortstam & Ryvarden	UC2023169	KP814365	–	Rosenthal et al. 2017	USA, Montana
	KHL8530 (GB)	–	AY586675	Larsson et al. 2004	Sweden
*X.astrocystidiatus* (Yurchenko & Sheng H.Wu) Riebesehl, Yurchenko & Langer	Wu 9211-71	JN129972	JN129973	Yurchenko and Wu 2014	Taiwan
***X.australis* (Berk.) Hjortstam & Ryvarden**	**CIEFAP-11041 (CFMR)**	n.a.	**MH884895**	**This study**	**Argentina**
*X.bisporus* (Boidin & Gilles) Hjortstam & Ryvarden	–	n.a.	n.a.	–	–
*X.borealis* (Kotir. & Saaren.) Hjortstam & Ryvarden	UC2022850	KP814307	–	Rosenthal et al. 2017	USA, Connecticut
	JS26064	–	AY586677	Larsson et al. 2004	Norway
*X.bresinskyi* (Langer) Hjortstam & Ryvarden	–	n.a.	n.a.	–	–
*X.brevisetus* (P. Karst.) Hjortstam & Ryvarden	KHL 12386 (GB)	DQ873612	DQ873612	Larsson et al. 2006	Sweden
*X.bubalinus* (Min Wang, Yuan Y. Chen & B.K. Cui) C.C. Chen & Sheng	CLZhao 184	MG231628	n.a.	unpublished	China
	Cui 6834	KY290981	–	Wang and Chen 2017	China
	Cui 12887	KY290982	–	Wang and Chen 2017	China
	Cui 12888, Holotype	KY290983	–	Wang and Chen 2017	China
*X.candidissimus* (Berk. & M.A.Curtis) Hjortstam & Ryvarden	–	n.a.	n.a.	–	–
*X.capitatus* (G.Cunn.) Hjortstam & Ryvarden	–	n.a.	n.a.	–	–
*X.chinensis* (C.C.Chen & Sheng H.Wu) C.C.Chen & Sheng H.Wu	Wu 1307-42	KX857802	–	Chen et al. 2017	China
	Wu 1407-105, Holotype	KX857804	KX857811	Chen et al. 2017	China
*X.crassihyphus* (Douanla-Meli) Riebesehl & Langer	–	n.a.	n.a.	–	–
*X.crassisporus* (Gresl. & Rajchenb.) Hjortstam & Ryvarden	–	n.a.	n.a.	–	–
*X.crustosoglobosus* (Hallenb. & Hjortstam) Hjortstam & Ryvarden	–	n.a.	n.a.	–	–
***X.cystidiatus* (A.David & Rajchenb.) Riebesehl & Langer**	**FR-0249200**	**MH880195**	**MH884896**	**This study**	**Réunion**
*X.detriticus* (Bourdot) Tura, Zmitr., Wasser & Spirin	UC2023108	KP814412	n.a.	Rosenthal et al. 2017	USA, Michigan
*X.echinatus* (Yurchenko & Sheng H.Wu) Riebesehl, Yurchenko & Langer	–	n.a.	n.a.	–	–
*X.erikssonii* (M.Galán & J.E.Wright) Riebesehl & Langer	–	n.a.	n.a.	–	–
***X.exilis* Yurchenko, Riebesehl & Langer**	**MSK-F 7381**	**MH880196**	–	**This study**	**Taiwan**
	**MSK-F 7431**	–	**MH884897**	**This study**	**Taiwan**
	**TUB-FO 42450**	**MH880197**	–	**This study**	**Taiwan**
	**TUB-FO 42565, Holotype**	**MH880198**	**MH884898**	**This study**	**Taiwan**
***X.filicinus* Yurchenko & Riebesehl**	**MSK-F 12869, Holotype**	**MH880199**	**MH884899**	**This study**	**Taiwan**
	**MSK-F 12870**	**MH880200**	**MH884900**	**This study**	**Taiwan**
*X.fimbriatus* (Sheng H.Wu) Hjortstam & Ryvarden	–	n.a.	n.a.	–	–
***X.flaviporus* (Berk. & M.A.Curtis ex Cooke) Riebesehl & Langer**	FCUG 1053	AF145575	–	Paulus et al. 2000	Romania
	**FR-0249797**	**MH880201**	**MH884901**	**This study**	**Réunion**
	**KAS-GEL3462**	**MH880202**	–	**This study**	**Taiwan**
	**KAS-GEL5047**	**MH880203**	–	**This study**	**Réunion**
	KUC20130808-17	–	KJ668314	Jang et al. 2016	South Korea
***X.follis* Riebesehl, Yurchenko & Langer**	**FR-0249814, Holotype**	**MH880204**	**MH884902**	**This study**	**Réunion**
*X.gamundiae* (Gresl. & Rajchenb.) Riebesehl & Langer	–	n.a.	n.a.	–	–
*X.gracilis* (Hjortstam & Ryvarden) Hjortstam & Ryvarden	–	n.a.	n.a.	–	–
*X.hallenbergii* (Sheng H.Wu) Hjortstam & Ryvarden	–	n.a.	n.a.	–	–
*X.hastifer* (Hjortstam & Ryvarden) Hjortstam & Ryvarden	Ryvarden 19767, Holotype	KY081801	n.a.	Riebesehl and Langer 2017	Argentina
*X.heterocystidiatus* (H.X.Xiong, Y.C.Dai & Sheng H.Wu) Riebesehl, Yurchenko & Langer	Wu 9209-27	JX175045	–	Yurchenko and Wu 2014	Taiwan
	–	–	KX857821	Chen et al. 2017	–
*X.hjortstamii* (Gresl. & Rajchenb.) Riebesehl & Langer	–	n.a.	n.a.	–	–
***X.hyphodontinus* (Hjortstam & Ryvarden) Riebesehl, Yurchenko & G.Gruhn**	**KAS-GEL9222**	**MH880205**	**MH884903**	**This study**	**Kenya**
	**LIP GG-GUY13-044**	**MH880206**	**MH884904**	**This study**	**French Guyana**
	**LIP GG-MAR12-238**	**MH880207**	**MH884905**	**This study**	**Martinique**
	**LIP GG-MAR15-127**	**MH880208**	**MH884906**	**This study**	**Martinique**
*X.knysnanus* (Van der Byl) Hjortstam & Ryvarden	–	n.a.	n.a.	–	–
*X.lanatus* (Burds. & Nakasone) Hjortstam & Ryvarden	–	n.a.	n.a.	–	–
*X.lenis* Hjortstam & Ryvarden	Wu0808-32	–	KX857820	Chen et al. 2017	Taiwan
	Wu890714-3, Holotype	KY081802	–	Riebesehl and Langer 2017	Taiwan
*X.lutescens* (Hjortstam & Ryvarden) Hjortstam & Ryvarden	–	n.a.	n.a.	–	–
*X.mollissimus* (L.W.Zhou) C.C.Chen & Sheng H.Wu	LWZ20160318-3	KY007517	n.a.	Kan et al. 2017	China
*X.mussooriensis* Samita, Sanyal & Dhingra	–	n.a.	n.a.	–	–
***X.nespori* (Bres.) Hjortstam & Ryvarden**	KAS-GEL3158	–	DQ340346	unpublished	Sweden
	**KAS-JR14**	**MH880210**	–	**This study**	**Germany**
	KUC20161012-50	MF774797	–	unpublished	South Korea
*X.nesporina* (Hallenb. & Hjortstam) Hjortstam & Ryvarden	–	n.a.	n.a.	–	–
***X.niemelaei* (Sheng H.Wu) Hjortstam & Ryvarden**	Dai 15358	KT989973	–	Chen et al. 2016	China
	**FR-0219860**	**MH880211**	–	**This study**	**Réunion**
	**FR-0249174**	**MH880212**	–	**This study**	**Réunion**
	**FR-0249178**	–	**MH884907**	**This study**	**Réunion**
	**FR-0249225**	**MH880213**	–	**This study**	**Réunion**
	**FR-0249289**	**MH880214**	–	**This study**	**Réunion**
	**FR-0249744**	**MH880215**	–	**This study**	**Réunion**
	**FR-0249811**	**MH880216**	–	**This study**	**Réunion**
	**FR-0249846**	**MH880217**	–	**This study**	**Réunion**
	GC 1508-146	KX857798	–	Chen et al. 2017	Taiwan
	**KAS-GEL4904**	**MH880218**	–	**This study**	**Réunion**
	KAS-GEL4998	EU583422	–	unpublished	Réunion
	Wu1010-62	–	KX857817	Chen et al. 2017	Taiwan
*X.nongravis* (Lloyd) C.C.Chen & Sheng H.Wu	GC1412-22	KX857801	KX857818	Chen et al. 2017	Taiwan
*X.nothofagi* (G.Cunn.) Hjortstam & Ryvarden	PDD:91630	GQ411524	–	Fukami et al. 2010	New Zealand
*X.nudisetus* (Warcup & P.H.B.Talbot) Hjortstam & Ryvarden	–	n.a.	n.a.	–	–
*X.ovisporus* (Corner) Riebesehl & Langer	ICMP 13830	AF145584	–	Paulus et al. 2000	New Zealand
	KAS-GEL3493	EU583421	–	unpublished	Taiwan
	KUC20130725-29	–	KJ668365	Jang et al. 2016	South Korea
*X.papillosus* (Fr.) Riebesehl, Yurchenko & Langer	–	n.a.	n.a.	–	–
***X.paradoxus* (Schrad.) Chevall.**	FCUG 2425	AF145571	–	Paulus et al. 2000	Russia
	KAS-GEL2511	–	AF518647	Hibbett and Binder 2002	Germany
	**KAS-JR06**	**MH880219**	–	**This study**	**Germany**
	**KAS-JR28**	–	**MH884908**	**This study**	**Austria**
*X.pelliculae* (H.Furuk.) Riebesehl, Yurchenko & Langer	–	n.a.	n.a.	–	–
*X.poroideoefibulatus* (Sheng H.Wu) Hjortstam & Ryvarden	–	n.a.	n.a.	–	–
*X.pruniaceus* (Hjortstam & Ryvarden) Hjortstam & Ryvarden	–	n.a.	n.a.	–	–
***X.pseudolanatus* Nakasone, Yurchenko & Riebesehl**	**FP-150922 (CFMR), Holotype**	**MH880220**	**MH884909**	**This study**	**Belize**
*X.pseudotropicus* (C.L.Zhao, B.K.Cui & Y.C.Dai) Riebesehl, Yurchenko & Langer	Dai 10768	KF917543	n.a.	Zhao et al. 2014	China
*X.quercinus* (Pers.) Gray	Otto Miettinen 15050,1 (H 6013352)	KT361632	–	Ariyawansa et al. 2015	Finland
	KHL11076 (GB)	KT361633	AY586678	Larsson et al. 2004	Sweden
***X.raduloides* (Pers.) Riebesehl & Langer**	ICMP 13833	AF145580	–	Paulus et al. 2000	Australia
	**KAS-JR 02**	**MH880221**	–	**This study**	**Germany**
	**KAS-JR 03**	**MH880222**	–	**This study**	**Germany**
	**KAS-JR 09**	**MH880223**	–	**This study**	**Germany**
***X.raduloides* (Pers.) Riebesehl & Langer**	**KAS-JR 10**	**MH880224**	–	**This study**	**Germany**
	**KAS-JR 26**	**MH880225**	**MH884910**	**This study**	**Germany**
	**LR 18813**	**MH880226**	**MH884911**	**This study**	**Australia**
*X.ramicida* Spirin & Miettinen	Viacheslav Spirin 7664 (H), Holotype	KT361634	n.a.	Ariyawansa et al. 2015	Russia
*X.reticulatus* (C.C.Chen & Sheng H.Wu) C.C.Chen & Sheng H.Wu	GC1512-1	KX857808	KX857813	Chen et al. 2017	Taiwan
	KUC20160721B-26	MF774798	–	Kwon et al. 2018	South Korea
	Wu1109-178, Holotype	KX857805	–	Chen et al. 2017	Taiwan
*X.rhizomorphus* (C.L.Zhao, B.K.Cui & Y.C.Dai) Riebesehl, Yurchenko & Langer	Dai 12354	KF917544	n.a.	Zhao et al. 2014	China
	Dai 12367, Holotype	KF917545	–	Zhao et al. 2014	China
	Dai 12389	KF917546	–	Zhao et al. 2014	China
*X.rickii* (Hjortstam & Ryvarden) K.H. Larss.	–	n.a.	n.a.	–	–
*X.rimosissimus* (Peck) Hjortstam & Ryvarden	Ryberg 021031 (GB)	DQ873627	DQ873628	Larsson et al. 2006	Sweden
*X.rudis* (Hjortstam & Ryvarden) Hjortstam & Ryvarden	–	n.a.	n.a.	–	–
*X.septocystidiatus* (H.X.Xiong, Y.C.Dai & Sheng H.Wu) Riebesehl & Langer	–	n.a.	n.a.	–	–
***X.serpentiformis* (Langer) Hjortstam & Ryvarden**	**KAS-GEL3668**	**MH880227**	–	**This study**	**Taiwan**
	**TUB-FO 40675**	**MH880228**	–	**This study**	**Taiwan**
	**TUB-FO 40985**	–	**MH884912**	**This study**	**Taiwan**
	**TUB-FO 42688**	**MH880229**	**MH884913**	**This study**	**Taiwan**
*X.* sp. 1	Dai 15321	KT989969	n.a.	Chen et al. 2016	China
***X.spathulatus* (Schrad.) Kuntze**	KAS-GEL2690	KY081803	–	Riebesehl and Langer 2017	Germany
	**KAS-MMS7224**	**MH880230**	–	**This study**	**Czech Republic**
	KHL7085 (GB)	KY081804	–	Riebesehl and Langer 2017	Sweden
	**MSK-F 12931**	**MH880231**	**MH884914**	**This study**	**Russia**
***X.subclavatus* (Yurchenko, H.X.Xiong & Sheng H.Wu) Riebesehl, Yurchenko & Langer**	**TUB-FO 42167**	**MH880232**	n.a.	**This study**	**Taiwan**
***X.subflaviporus* C.C.Chen & Sheng H.Wu**	**KAS-GEL3466**	**MH880233**	–	**This study**	**Taiwan**
	Wu 0809-76	KX857803	KX857815	Chen et al. 2017	China
*X.subglobosus* Samita, Sanyal & Dhingra	–	n.a.	n.a.	–	–
*X.submucronatus* (Hjortstam & Renvall) Hjortstam & Ryvarden	–	n.a.	n.a.	–	–
*X.subscopinellus* (G.Cunn.) Hjortstam & Ryvarden	–	n.a.	n.a.	–	–
*X.subtropicus* (C.C.Chen & Sheng H.Wu) C.C.Chen & Sheng H.Wu	Wu 1508-2	KX857806	KX857812	Chen et al. 2017	China
	Wu 9806-105, Holotype	KX857807	KX857809	Chen et al. 2017	Vietnam
*X.syringae* (Langer) Hjortstam & Ryvarden	–	n.a.	n.a.	–	–
*X.taiwanianus* (Sheng H.Wu) Hjortstam & Ryvarden	–	n.a.	n.a.	–	–
*X.tenellus* Hjortstam & Ryvarden	–	n.a.	n.a.	–	–
*X.tenuicystidius* (Hjortstam & Ryvarden) Hjortstam & Ryvarden	–	n.a.	n.a.	–	–
*X.trametoides* (Núñez) Riebesehl & Langer	–	n.a.	n.a.	–	–
*X.tuberculatus* (Kotir. & Saaren.) Hjortstam & Ryvarden	–	n.a.	n.a.	–	–
*X.verecundus* (G.Cunn.) Yurchenko & Riebesehl	KHL 12261 (GB)	DQ873642	n.a.	Larsson et al. 2006	USA
*X.vesiculosus* Yurchenko, Nakasone & Riebesehl	–	n.a.	n.a.	–	–

### Morphology

#### Xylodon exilis

Taxon classificationFungiHymenochaetalesSchizoporaceae

Yurchenko, Riebesehl & Langer sp. nov.

MB827462

Figs 3a , 4

##### Holotype.

TAIWAN, Nantou county, south from Sun-Moon Lake, near Hua Lien, Lien-Hwa-Chi, 700 m a.s.l., on fallen angiosperm twig, leg. E. Langer, G. Langer, F. Oberwinkler, 10 Jul 1990 (TUB-FO 42565; isotypes in KAS and MSK).

##### Description.

Basidiomata effused, 1–5 cm in extent, membranaceous, discontinuous at the periphery. Hymenial surface minutely odontioid, cream-coloured, between aculei 50–130 μm thick. Aculei peg-like, conical or subcylindrical, entire or slightly penicillate apically, 35–70 μm long, 15–50(–70) μm diam., 8–14/mm. Margin abrupt or somewhat thinning out. Hyphal system monomitic, hyphae colourless, with clamps at all primary septa. Subicular hyphae forming a loose tissue, rarely branched, 3–4(–4.5) μm wide, with slightly thick to thick walls (0.5–1.2 μm thick), with scattered adventitious septa, smooth. Subhymenial hyphae in a dense tissue, richly branched, 2–3 μm wide, thin-walled, smooth or slightly encrusted. Capitate cystidia enclosed, 18–22 × 5.5–8 μm, sometimes with an adventitious septum in stem, thin- to slightly thick-walled. Projecting hyphae in aculei flexuous, apically obtuse, 90–130 μm long, 3–4 μm wide, originating from thick-walled subicular hyphae, with simple and clamped septa, often constricted at septa, walls thickened at base then gradually thinning toward apex, moderately encrusted. Basidioles clavate or bowling pin-shaped, 10–20 × 4.5–5.5 μm. Basidia narrowly utriform, 20–25 × 4–5(–5.5) μm, thin-walled, smooth, with four sterigmata 2–4 × 0.3 μm. Spores narrowly ellipsoid, 5.5–6 × 2.5–3 μm, holotype L = 5.8 µm, W = 2.8 µm, Q = (1.6–)1.8–2.2, colourless, smooth, slightly thick-walled, negative in Mz, acyanophilous, with minute apiculus.

**Figure 3.**
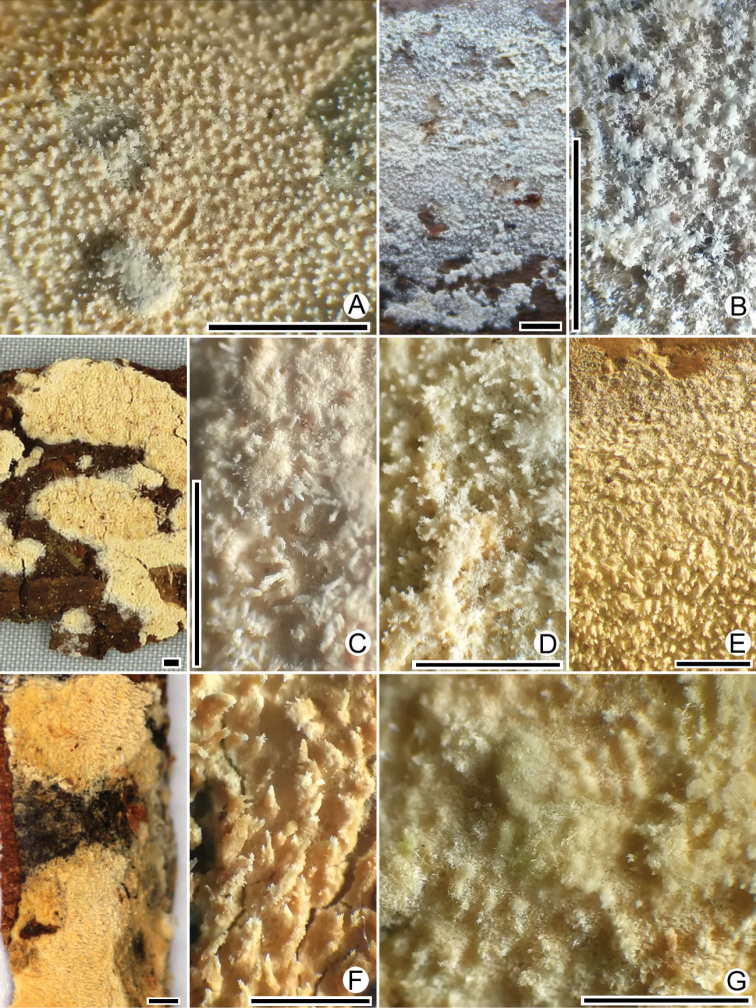
Basidiomata of *Xylodon* spp. **A***X.exilis* (TUB-FO 42565, holotype) **B***X.filicinus* (MSK-F 12369, holotype) **C***X.follis* (FR-0249814, holotype) **D***X.pseudolanatus* (FP-150922, holotype) **E***X.pseudolanatus* (HHB-6925, paratype) **F***X.vesiculosus* (PDD-18112, isotype) **G***X.lanatus* (CFMR HHB-8925, holotype). Scale bars: 1 mm.

##### Distribution and ecology.

The species is known from Taiwan and Nepal. It grows on dead wood of angiosperms, with a preference for small branches and twigs.

##### Etymology.

from Latin *exilis* – thin, fine, refers to the small and narrow aculei.

**Figure 4.**
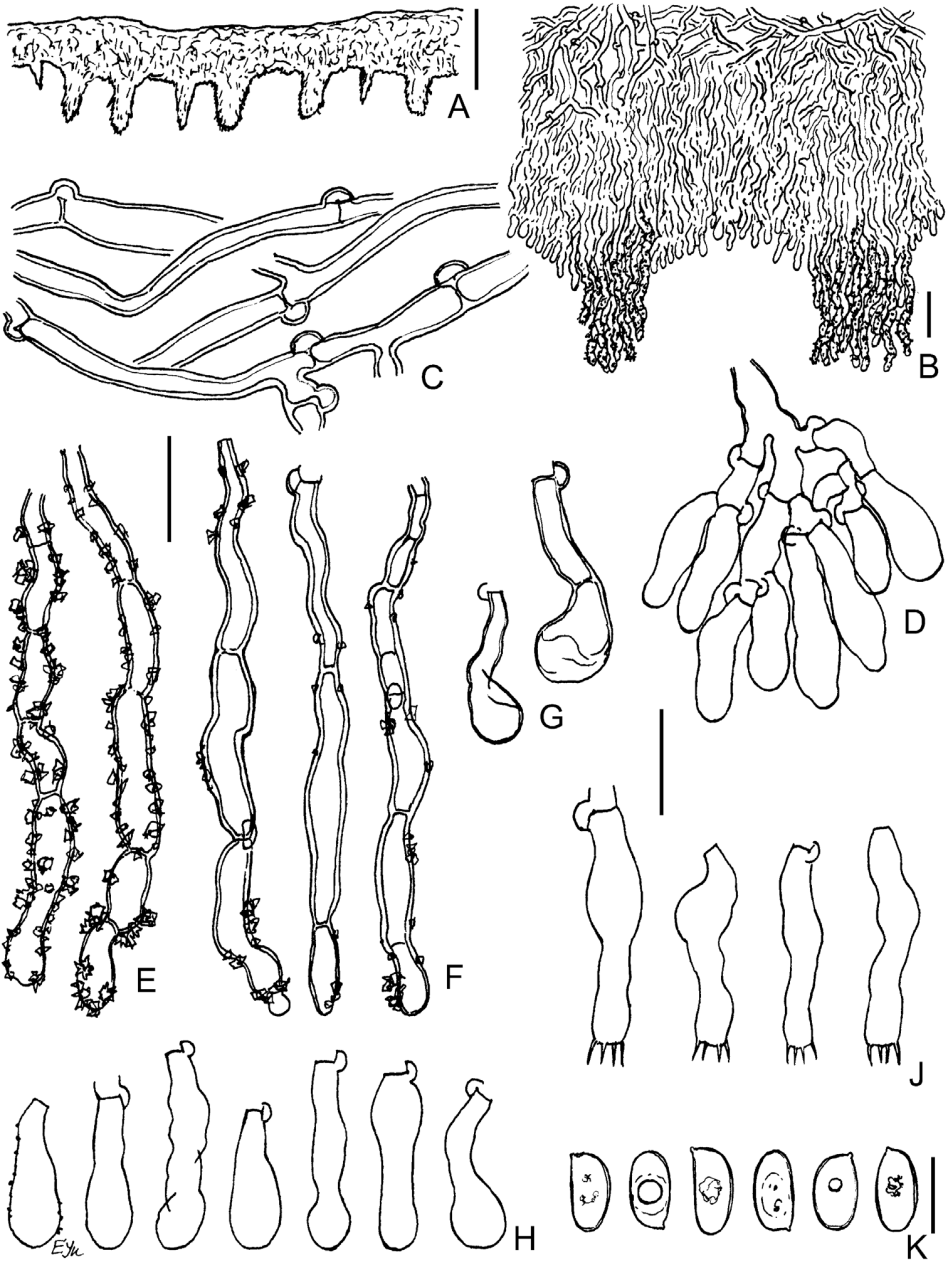
Micromorphology of *Xylodonexilis* (TUB-FO 42565, holotype): **A, B** vertical sections through basidioma **C** subicular hyphae **D** portion of hymenium and subhymenium **E** projecting aculeal hyphae in Mz**F** projecting aculeal hyphae in 3% KOH**G** capitate cystidia **H** basidioles **J** basidia **K** basidiospores. Scale bars: 100 μm (**A**); 20 μm (**B**); 10 μm (**C–J**); 5 μm (**K**).

##### Additional specimens examined.

TAIWAN, Nantou Co., west from Sun-Moon Lake, near Hua Lien, on dead wood, leg. E. Langer, G. Langer, F. Oberwinkler, 26 Mar 1989 (TUB-FO 40734; dupl. in KAS); south from Sun-Moon Lake, near Hua Lien, on fallen angiosperm twig, leg. E. Langer, G. Langer, F. Oberwinkler, 9 Jul 1990 (TUB-FO 42450; dupl. in KAS and MSK); Taichung Co., Shinshe, on fallen angiosperm twig, leg. E. Yurchenko, 2 Apr 2011 (MSK-F 12912); ibid., on fallen liana stem, leg. E. Yurchenko, 2 Apr 2011 (MSK-F 12913); ibid., on fallen angiosperm twig, leg. E. Yurchenko, 5 Jun 2011 (MSK-F 12914); Taipei Co., Wulai, Neidong Recreation Area, on fallen angiosperm twig, leg. E. Yurchenko, 23 Jun 2011 (MSK-F 7381; dupl. in KAS and LE); Miaoli Co., Sanyi, on fallen angiosperm branch, leg. E. Yurchenko, 3 Jul 2011 (MSK-F 7431); ibid., on fallen angiosperm branch, leg. E. Yurchenko, 19 Jul 2011 (MSK-F 7430). NEPAL: Gandaki Prov., Kuldi, Anapurna Trek, leg. L. Ryvarden, 7 Nov 1979 (O-LR 18918/B, dupl. in KAS).

##### Remarks.

The species concept of *X.lanatus* is revised and restricted to specimens with a well-developed woolly subiculum. The distinctive characters of *X.exilis* are the minutely odontioid basidiomata with peg-like aculei composed of flexuous, encrusted, septate projecting hyphae that are constricted at the septa, embedded capitate cystidia and narrowly ellipsoid spores with slightly thickened walls. Earlier specimens of *X.exilis* from Taiwan (e.g. Langer 1994, p. 143 for illustration) and Nepal (Hjortstam and Ryvarden 1984) were originally identified as *X.lanatus*. Hyphae and spores of this species were also depicted by Yurchenko et al. (2013) under the name *X.lanatus*. These two species and other morphologically similar taxa are compared in the Discussion section below; a key is also presented.

#### Xylodon filicinus

Taxon classificationFungiHymenochaetalesSchizoporaceae

Yurchenko & Riebesehl sp. nov.

MB827463

Figs 3b , 5

##### Holotype.

TAIWAN, Nantou Co., Xitou (Shitou) Forest Recreation Area, W slope of Phoenix Mt. Range, 1470 m a.s.l., 23°40'N, 120°48'E, old-growth sparse broadleaf forest, on dead detached rachis of *Cyathea* sp., leg. E. Yurchenko, 31 Jul 2011 (field No. 38; MSK-F 12869; isotype in KAS).

##### Description.

Basidiomata effused, white, 2–4 cm in extent, farinaceous or pruinose, very loose or discontinuous, odontioid, 30–55 μm thick between aculei. Margin thinning out. Aculei conical or subcylindrical, 40–80 μm long, 15–45 μm diam., peg-like, of loose texture, 8–14/mm. Hyphal system monomitic, hyphae colourless, clamped at all septa. Subicular hyphae in a loose tissue, rarely branched, 2–3 μm diam., thin- or slightly thick-walled, loosely encrusted, under the subhymenium with inflations 5–6.5 μm wide. The largest crystals in subiculum 6–8 μm across, aggregated in clusters 15–18 μm diam. Subhymenial hyphae moderately branched, partly short-celled and slightly inflated, 2–3.5(–4) μm diam., lightly encrusted. Projecting hyphae in aculei richly encrusted, 20–45 × 5–7 μm in encrusted part, with clamped and simple septa, basally thick-walled, then becoming thin-walled, obtuse, sometimes subacute at apex. Cystidia in hymenium thin-walled, lightly encrusted, of three types: (1) subcylindrical, often slightly tapered to apex, numerous, 20–35 × 4.5–5.5 μm; (2) capitate, rare, 26–32 μm long, 3–5 μm wide at base, 2.5–3 μm wide at apex; (3) hyphoid to narrowly ventricose, about 30 × 4.5 μm. Basidioles ellipsoid, ovoid, clavate, 7.5–18 × 4.5–7.5 μm, more or less encrusted. Basidia utriform, (14–)16–20 × (3.5–)4.5–5.5 μm, thin-walled, smooth or sparsely encrusted, with four sterigmata 2–6.5 × 1–1.5 μm. Spores globose to subglobose, 4–5(–5.5) × (3.7–)4–4.5 μm, holotype L = 4.7 µm, W = 4.1 µm, Q = 1.1–1.2, thin-walled, often with one large oil-like globule, negative in Mz, weakly cyanophilous, with minute apiculus.

**Figure 5.**
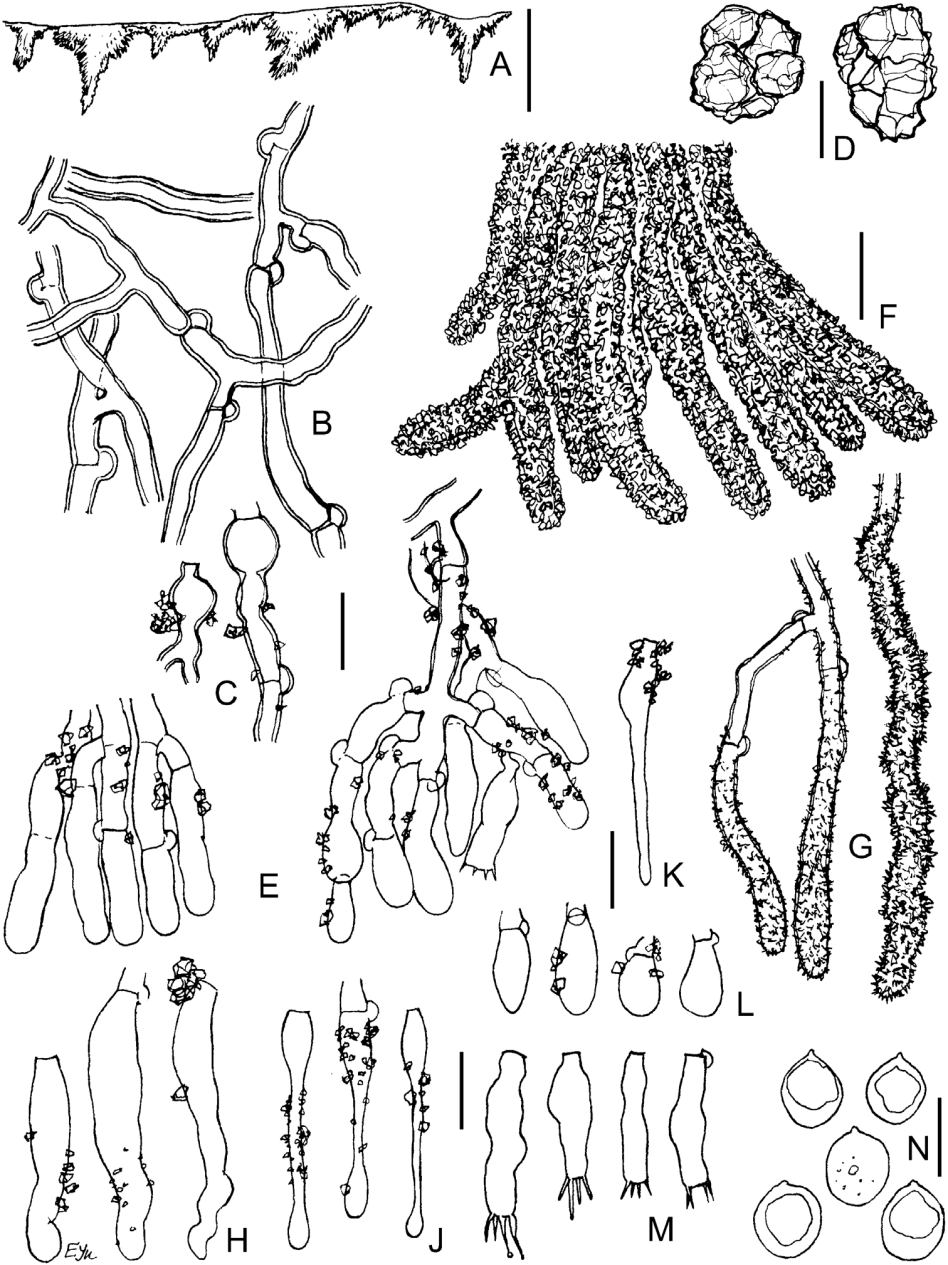
Micromorphology of *Xylodonfilicinus* (MSK-F 12369, holotype): **A** vertical section through basidioma **B** subicular hyphae **C** inflations on hyphae in lower subhymenium **D** crystals from subiculum **E** portions of hymenium and subhymenium **F** bundle of encrusted projecting hyphae **G** separate projecting hyphae **H** subcylindrical cystidia **J** capitate cystidia **K** hyphoid cystidium **L** basidioles **M** basidia **N** basidiospores. Scale bars: 100 μm (**A**); 10 μm (**B–M**); 5 μm (**N**).

##### Distribution and ecology.

From the lower mountainous belt in Taiwan, on dead fern rachises.

##### Etymology.

from Latin *filix* ‒ fern, refers to the occurrence on dead fern rachises.

*Additional specimen examined*. TAIWAN, the same locality and the same substrate as holotype, leg. E. Yurchenko, 31 Jul 2011 (field No. 18; MSK-F 12870; dup. in KAS).

##### Remarks.

**Figure 6.**
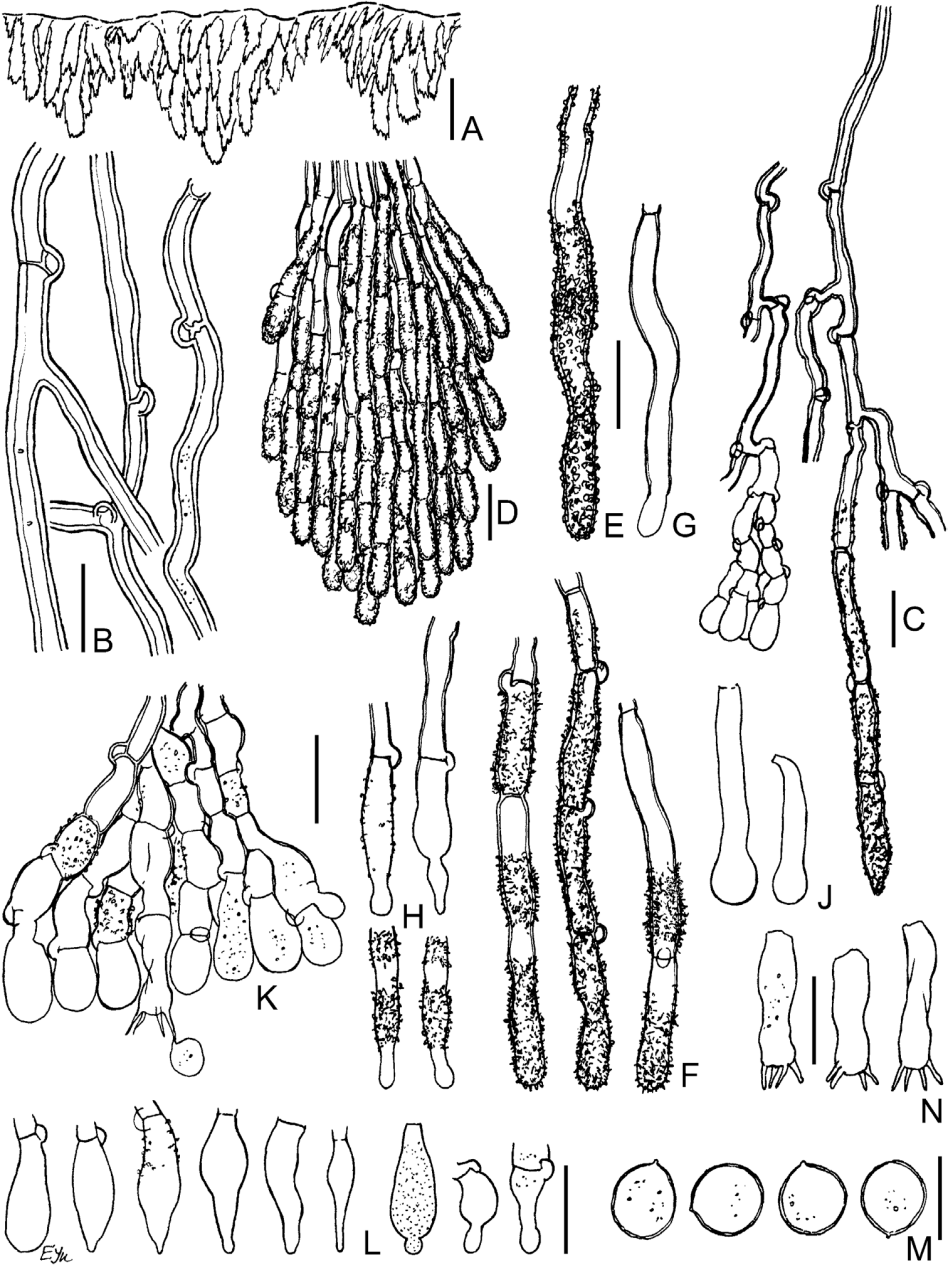
Micromorphology of *Xylodonhyphodontinus*. LIP GG-MAR 15-127: **A** vertical section through basidioma **B** subicular hyphae **C** excerpt of tramal hyphae to hymenium and projecting hyphae **D** bundle of encrusted aculeal hyphae **E** encrustation on projecting hypha in water **F** encrustation on projecting hyphae in 3% KOH**G** naked projecting hyphal end **H** variously shaped hyphal ends **J** capitate cystidia **K** portion of hymenium and subhymenium **L** basidioles and cystidioles **M** basidiospores. LIP GG-MAR 12-238: **N** basidia. Scale bars: 100 μm (**A**); 10 μm (**B–L, N**); 5 μm (**M**).

The distinctive features of this species are the pruinose, minutely odontioid basidiomata, fascicles of richly encrusted projecting hyphae in aculei and the three types of cystidia. *Xylodonfilicinus* is morphologically similar to *X.hyphodontinus*, which differs in having projecting hyphae in the aculei that are straighter with more septa, a denser subhymenium composed of short-celled hyphae, short, ventricose cystidioles and spore walls that are slightly thickened at maturity (see Fig. 6).

#### Xylodon follis

Taxon classificationFungiHymenochaetalesSchizoporaceae

Riebesehl, Yurchenko & Langer sp. nov.

MB827464

Figs 3c , 7

##### Holotype.

REUNION, Forêt Notre Dame de la Paix, Sentier botanique, 21°15.8'S, 55°36.1'E, 1720 m a.s.l., on angiosperm wood, leg. J. Riebesehl, M. Schröder, M.M. Striegel, 12 Mar 2015 (FR-0249814; isotypes in KAS (as L1040) and MSK).

##### Description.

Basidiomata effused, cream-coloured, about 1–5 cm in extent, soft-membranaceous, continuous, finely aculeate, between aculei 50–200 μm thick; aculei narrowly conical or nearly cylindrical, 80–170 × 20–40(–60) μm, 10–12/mm, fragile, slightly fimbriate at apices, sterile. Margin abrupt. Hyphal system monomitic; hyphae clamped and simple septate, colourless, (1–)2–3.5 μm diam. Subiculum little differentiated, composed of thin- to slightly thick-walled hyphae. Hyphae in aculeal trama mostly parallel, thin- to thick-walled (walls up to 1 μm thick), projecting through aculeal apices and loosely encrusted with crystals about 1–3 μm long in KOH. Subhymenium thickening; subhymenial hyphae moderately branched, thin-walled, smooth. Capitate cystidia numerous, projecting and immersed, in subhymenium, hymenium and aculei, 17–30(–40) × 4.5–9 μm, with 1–2 adventitious septa in stalk, apical cap encased with resinous encrustation 6–12 μm wide, easily dissolving in KOH and Mz, unchanged in CBL. Hyphidial elements common in hymenium, 17–27 × 2.3–3.2 μm. Basidioles pyriform or ellipsoid, 17–28 × 8–12 μm, with granular contents, smooth or slightly encrusted. Basidia utriform or suburniform, thin-walled, smooth, 32–37 × 9–10 μm, with 4 sterigmata 4–6.5 × 1.3–2.3 μm. Spores globose to subglobose, colourless, with homogeneous or granular contents, smooth, inamyloid, indextrinoid, cyanophilous, thin-walled, (7.5–)8–9.5(–10) × 7–8.5 μm, holotype L = 8.6 μm, W = 7.6 μm, Q = 1.0–1.2, outer wall layer sometimes swelling in KOH and CBL, with rounded-triangular apiculus.

**Figure 7.**
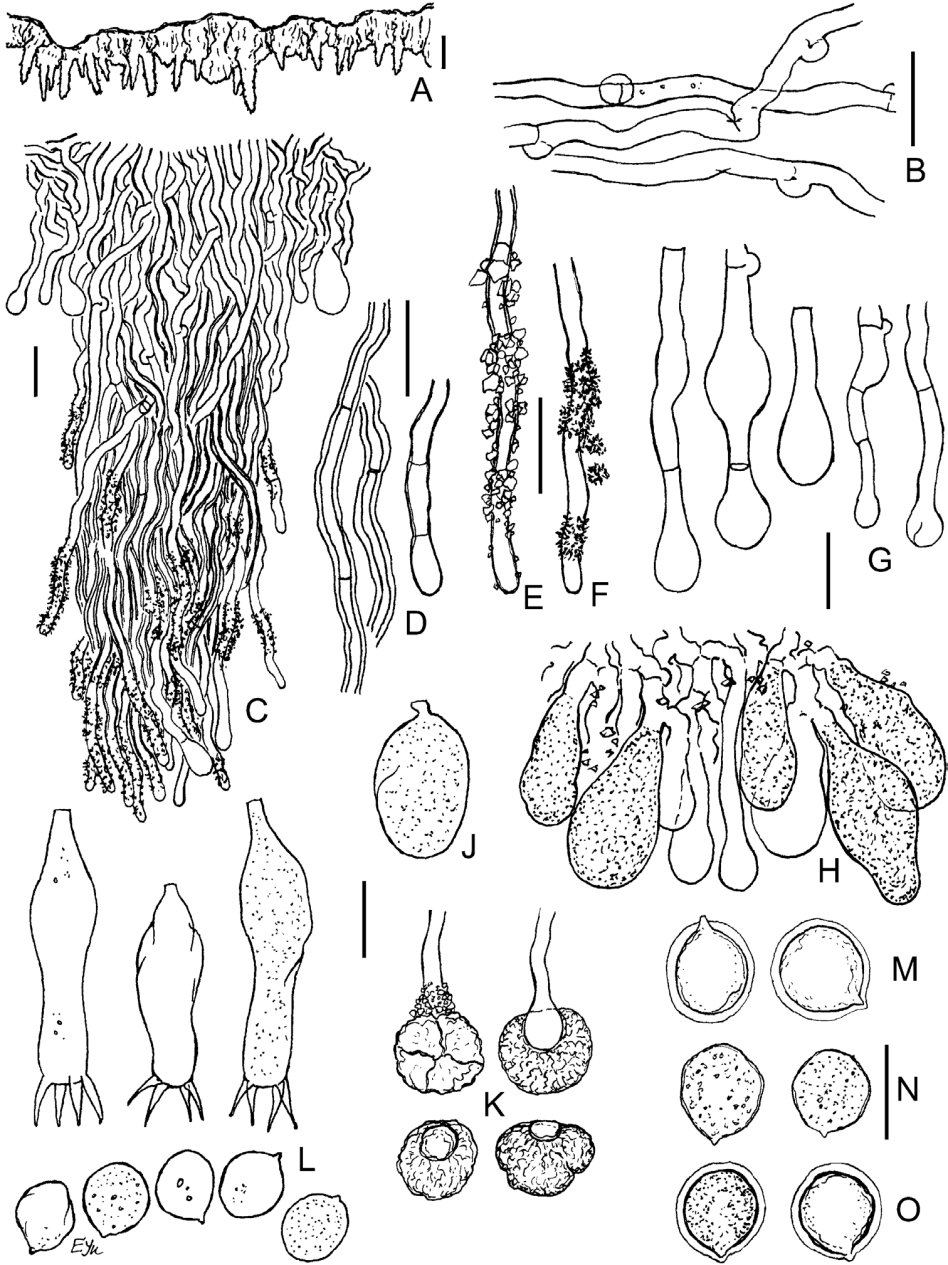
Micromorphology of *Xylodonfollis* (FR-0249814, holotype): **A** vertical section through basidioma **B** subicular hyphae **C** vertical section through aculeus **D** lower and apical part of aculeal hyphae with adventitious septa **E** encrusted aculeal hyphae in water **F** partially dissolved crystals on aculeal hyphae in 3% KOH**G** capitate cystidia **H** portion of hymenium **J** vesicular basidiole **K** capitate encrusted cystidia and their detached resinous caps **L** basidia and basidiospores **M** basidiospores in 3% KOH**N** basidiospores in water **O** basidiospores in CBL. Scale bars: 100 μm (**A**); 10 μm (**B–N**).

##### Distribution and ecology.

The species is so far known from Réunion (Mascarene Archipelago) and inhabits dead wood.

##### Etymology.

from Latin *follis* ‒ bag or bubble, referring to shape of the spores, basidioles and capitate cystidia found in this species.

*Additional specimen examined*. REUNION, Forêt de Bébour, 1328 m asl., leg. E. Langer, G. Langer, E. Hennen, 20 Mar 1998 (KAS-GEL 4951; dupl. in MSK).

##### Remarks.

This taxon differs from other *Xylodon* species by its unusually large basidia, large globose basidiospores with walls that swell in KOH and CBL and numerous simple septa as well as clamps on the hyphae. The swelling of spore walls was observed in some spores; spores were unaffected in water mounts. The hymenium has a granular appearance visible under 100× magnification because of the resinous cap developed on the capitate cystidia. The resinous caps are observed only in CBL and are easily detaching in squash preparations. Intermediate forms in morphology of hyphidia to capitate cystidia and of capitate cystidia to pyriform basidioles were frequently observed.

#### Xylodon pseudolanatus

Taxon classificationFungiHymenochaetalesSchizoporaceae

Nakasone, Yurchenko & Riebesehl sp. nov.

MB827465

Figs 3d , 8

##### Holotype.

BELIZE: Cayo District, Mountain Pine Ridge, on corticated hardwood branch, leg. K.K. Nakasone, 24 Nov 2001 (CFMR FP-150922; isotypes in KAS and MSK; ex-type culture CFMR FP-150922-sp; ex-type ITS sequence MH880220; ex-type 28S sequence MH884909).

##### Description.

Basidiomata effused, membranaceous, cream-coloured, 1–6 cm in extent, odontioid with conical aculei 50–120 μm long and 25–65 μm diam. at base, 8–14 aculei/mm. Subiculum between aculei very loose, minutely porulose, 100–150 μm thick. Margin pale cream-coloured, abrupt or diffuse, up to 2 μm wide. Hyphal system monomitic, hyphae clamped at all primary septa, colourless. Subicular hyphae little branched, mostly thick-walled, 2.5–4 μm diam., smooth or scarcely encrusted. Subhymenial hyphae richly branched, thin-walled, 2–3.5(–4.5) μm diam., smooth or slightly encrusted. Aculei consisting mostly of projecting hyphae. Projecting hyphae moderately flexuous, (3–)3.5–5 μm diam., slightly thick-walled, loosely encrusted, clamped at septa. Capitate cystidial elements found mostly in subhymenium and subiculum, scattered to frequent, terminal or lateral, smooth, thin- to thick-walled, aseptate or with adventitious septa, (8–)15–30 × (4.5–)5–6.5(–8.5) μm. Basidioles clavate to subcylindrical, sometimes slightly tapering to apex, 7–22 × 4–5 μm. Basidia cylindrical, sometimes slightly constricted, 16–30 × 4–4.3 μm, thin-walled, smooth, with four sterigmata about 2.5 × 0.2 μm. Spores narrowly ellipsoid to oblong, 5–6(–6.3) × (2.5–)3–3.5 μm, holotype L = 5.5 μm, W = 3.2 μm, Q = 1.7, thin- or slightly thick-walled, colourless, smooth, with minute apiculus, inamyloid, indextrinoid, weakly cyanophilous.

**Figure 8.**
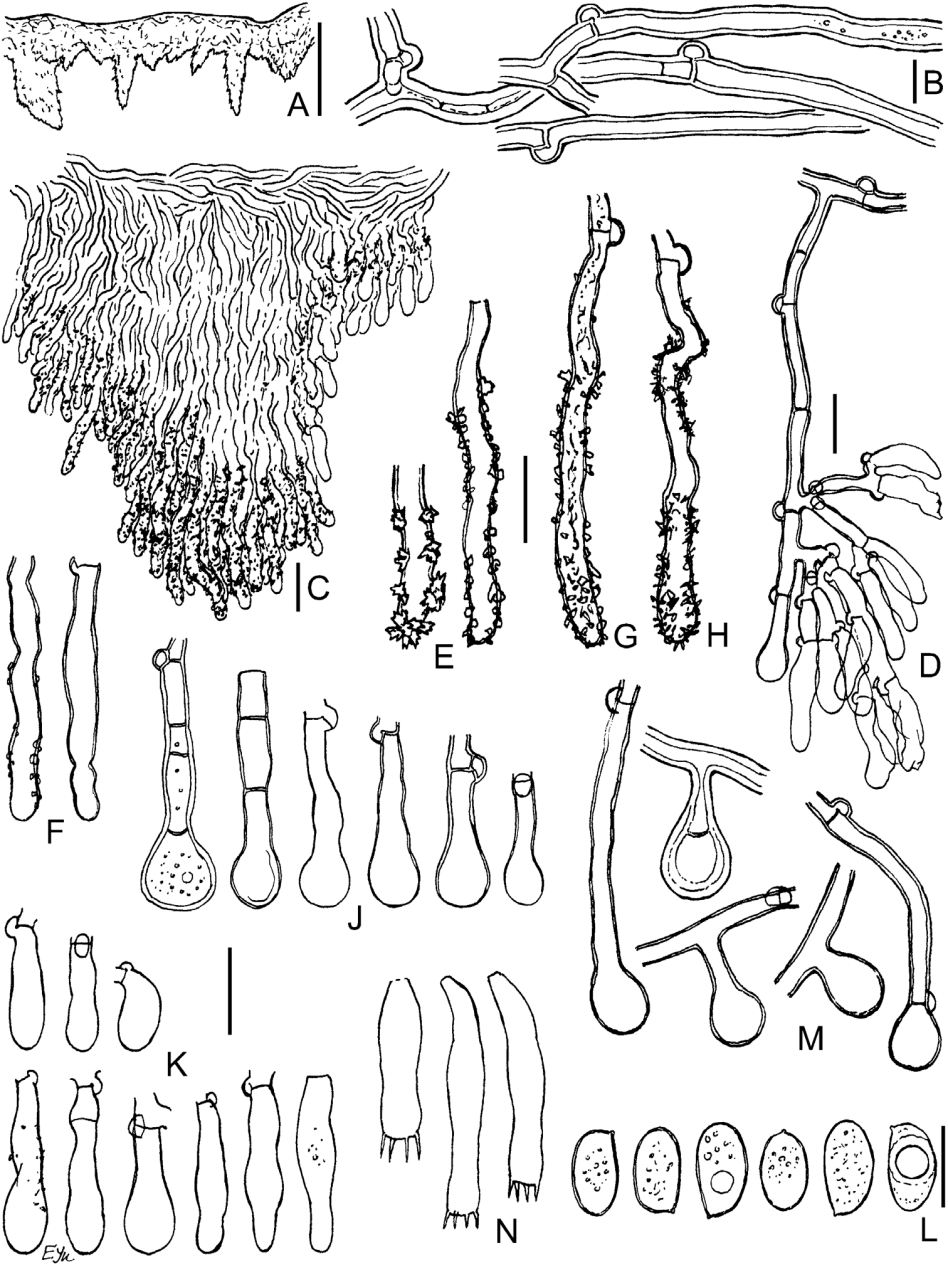
Micromorphology of *Xylodonpseudolanatus*. CFMR: FP-150922 (holotype): **A** vertical section through basidioma **B** subicular hyphae **C** vertical section through aculeus **D** detail of subicular hyphae and hymenium **E, F** projecting aculeal hyphae in 3% KOH**G** projecting aculeal hyphae in water **H** projecting aculeal hyphae in Mz**J** capitate cystidia in hymenium **K** basidioles **L** basidiospores. CFMR: HHB-6925: **M** capitate cystidia in subiculum **N** basidia. Scale bars: 100 μm (**A**); 10 μm (**C–K, M, N**); 5 μm (**B, L**).

##### Distribution and ecology.

South-eastern USA and Central America, on dead wood of angiosperms.

##### Etymology.

From Greek *pseudo*- – false, refers to its similarity to *X.lanatus*.

##### Additional specimens examined.

USA: Alabama, Escambia County, 3 miles east of Flomaton, on bark of *Taxodiumdistichum* (L.) Rich. (CFMR FP-103492), on bark of *Quercus* sp. (CFMR FP-103500), leg. A.S. Rhoades, 1 Nov 1952; Florida, Marion County, Okalawaha River, on dead inflorescence of *Sabalpalmetto* (Walter) Lodd. ex Schult. & Schult. f., leg. H.H. Burdsall, Jr., 3 Aug 1972 (CFMR HHB-6925; dupl. in KAS and MSK); Louisiana, Baton Rouge, on *Meliaazedarach* L., leg. C.J. Humphrey & C.W. Edgerton, 29 Aug 1909 (CFMR FP-5519).

##### Remarks.

The diagnostic features of this species are the minutely odontioid hymenophore, bundles of sparsely to moderately encrusted hyphae, projecting from aculeal apices, embedded capitate cystidia, cylindrical basidia and narrowly ellipsoid basidiospores. Some hymenial elements in this species are intermediate in morphology between basidioles, capitate cystidia and hyphal ends. *Xylodonpseudolanatus* can be distinguished from similar species in the key below (see Discussion).

#### Xylodon hyphodontinus

Taxon classificationFungiHymenochaetalesSchizoporaceae

(Hjortstam & Ryvarden) Riebesehl, Yurchenko & G.Gruhn comb. nov.

MB827758

Fig. 6

Odontiopsis hyphodontina Hjortstam & Ryvarden, Mycotaxon 12(1): 180 (1980) (Basionym). Typus of O.hyphodontina: TANZANIA, Morogoro Prov., Morogoro distr., Uluguri Mts., Morning Side Res. sta. ca. 5 km S of Morogoro, substrate unknown, leg. L. Ryvarden, 24–26 Feb 1973 (O L. Ryvarden 10949 – holotype).
= Hydnumambiguum Berk. & Broome, Journal of the Linnean Society, Botany 14(73): 60 (1873). Typus of H.ambiguum: SRI LANKA, Central Province, on dead wood (Berkeley No. 974 – holotype).
= Odontiopsisambigua (Berk. & Broome) Hjortstam, Mycotaxon 28(1): 35 (1987).
= Pteridomycessphaericosporus Boidin, Lanq. & Gilles, Mycotaxon 16(2): 490 (1983).

##### Remarks.

This new combination is based on the phylogenetic analyses of the ITS and 28S sequences as well as morphological study of specimens, including the holotype of *O.hyphodontina*. Originally, the collections from Martinique and French Guyana were identified as *O.ambigua*, but the molecular data clearly show that these collections are embedded in *Xylodon* (Figs 1, 2). Although *H.ambiguum* is the oldest name for this taxon, it cannot be transferred to *Xylodon* because the name is preoccupied by *X.ambiguus* (Peck) Kuntze (= *Veluticepsambigua* (Peck) Hjortstam & Telleria). *Odontiopsisambigua*, *P.sphaericosporus* and *O.hyphodontina* were recognised as conspecific by Hjortstam (1987, 1991). *Odontiopsishyphodontina* is the next oldest name and is chosen to represent this taxon. As *O.hyphodontina* is also the type of *Odontiopsis* Hjortstam & Ryvarden, *Odontiopsis* concomitantly becomes a synonym of *Xylodon*.

The newly generated ITS and 28S sequences of *X.hyphodontinus* hold comparable positions in a clade that includes three distinct lineages in both phylogenetic trees (Figs 1, 2). Specimens KAS-GEL9222 from Kenya and LIP GG-GUY13-044 from French Guyana each represent distinct lineages from the third lineage of LIP GG-MAR15-127 and LIP GG-MAR12-238 from Martinique. As species in *Hyphodontia* s.l. can be readily distinguished with ITS or 28S sequences, these three lineages should result in the recognition of three different species. However, we were not able to identify any definite morphological differences amongst the lineages in comparison with the holotype material from Tanzania. Cultures are not available for these specimens, thus intercompatibility tests are not possible. As a result, we decided to treat all three lineages as *X.hyphodontinus* at this time.

#### Xylodon vesiculosus

Taxon classificationFungiHymenochaetalesSchizoporaceae

Yurchenko, Nakasone & Riebesehl nom. nov.

MB827759

Figs 3e , 9

##### Replaced synonym.

*Odontiavesiculosa* G. Cunn., Transactions and Proceedings of the Royal Society of New Zealand 86(1): 75 (1959) nom. inval.

##### Typus.

NEW ZEALAND: Otago, Alton Valley, Tuatapere, leg. J.M. Dingley, Feb 1954 (PDD-18112 – holotype).

Cunningham (1959) described this new taxon as *Odontiavesiculosa* G. Cunn. Earlier, *Odontiavesiculosa* Burt was used for another species (Povah 1929). Consequently, *Odontiavesiculosa* G. Cunn. is an illegitimate name and a new name is required for this taxon (see Art. 6.11, 7.4 and 58.1 in Turland et al. 2018).

Below is a description based on the isotype of *X.vesiculosus* (CFMR).

##### Description.

Basidiomata effused, odontioid, membranaceous, with a densely odontioid, ochraceous hymenial surface. Margin mostly abrupt, some parts thinning out. Hymenophoral aculei cylindrical to conical, acute apically, 130–350 μm long, 60–150 μm diam. at base, 4 per mm. Subiculum 100–150 μm thick, minutely cracking. Hyphal system monomitic; hyphae clamped at all primary septa. Subicular and tramal hyphae thick-walled (wall up to 1.5 μm), 2.5–4 μm wide, often with narrow lumen, smooth, colourless, looking faint yellowish in mass due to refractive walls. Subhymenium well developed; hyphae richly branched, thin- to slightly thick-walled, yellowish in mass. Aculei bearing skeletal-like, naked or poorly encrusted, immersed hyphal ends and variously encrusted, thick-walled, projecting hyphae in bunches, 3.5–5 μm wide. Capitate elements common, as lateral branches on tramal or subhymenial hyphae, (25–)30–55 × 6.5–10.5 μm, thin- to thick-walled, aseptate or with 1–2 adventitious septa. Basidioles clavate, subcylindrical, utriform. Basidia utriform to subcylindrical and clavate, 15–22 × 4–5 μm, thin-walled, smooth, with four sterigmata ca. 2 × 0.5 μm. Spores ellipsoid to narrowly ellipsoid or short cylindric, 5.3–6.3(–7) × 3–4 μm, holotype L = 5.9, W = 3.4, Q = 1.8 (n = 22), with adaxial side flat to convex, smooth, thin-walled, colourless, with minute apiculus, inamyloid, indextrinoid, acyanophilous.

##### Remarks.

**Figure 9.**
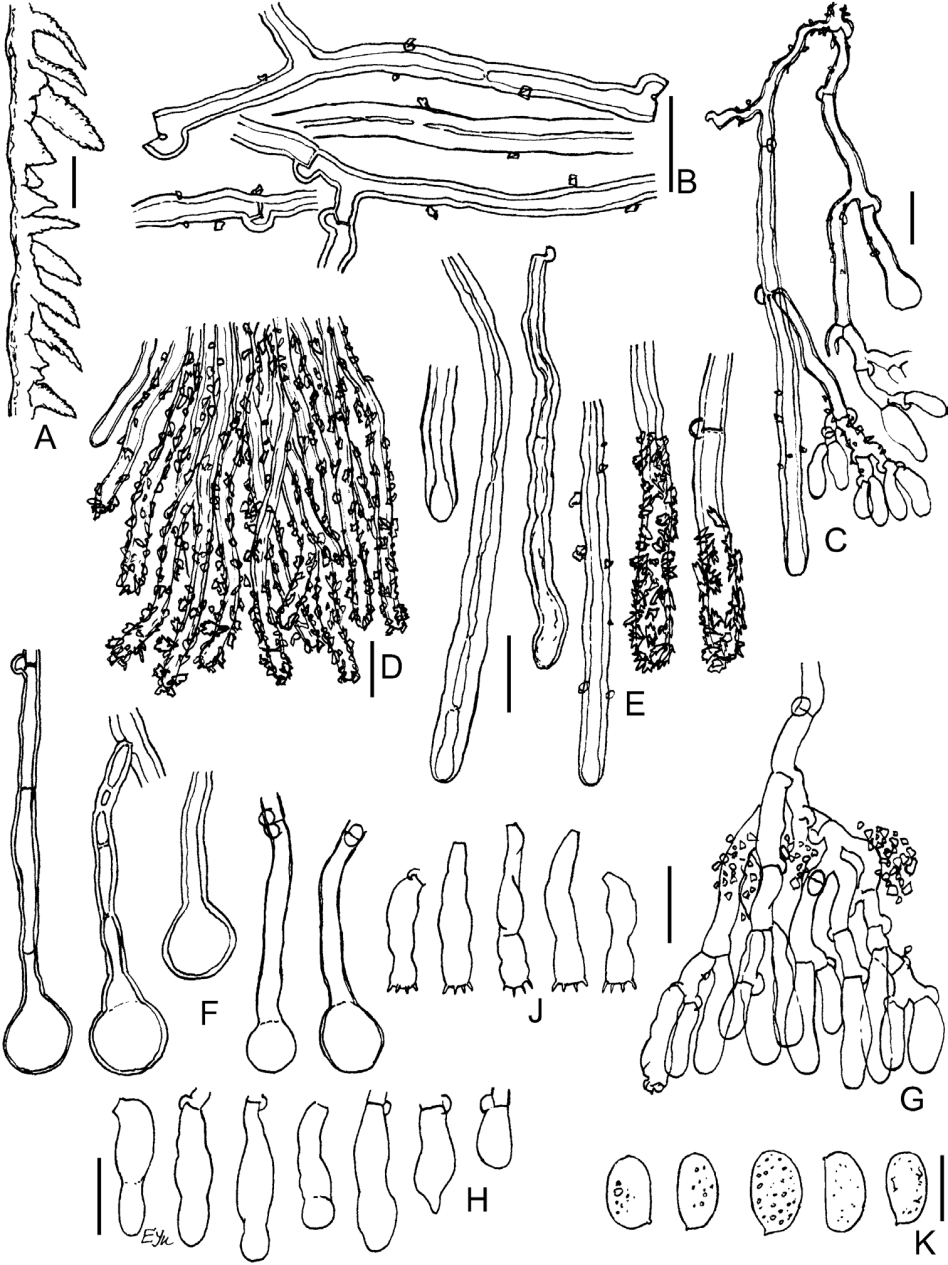
Micromorphology of *Xylodonvesiculosus* (PDD-18112, isotype): **A** vertical section through basidioma **B** subicular hyphae **C** excerpt of tramal hyphae to hymenium and skeletoid hyphae **D** bundle of projecting aculeal hyphae **E** smooth and variously encrusted aculeal hyphae **F** capitate cystidia **G** portion of hymenium and subhymenium **H** basidioles **J** basidia **K** basidiospores. Scale bars: 250 µm (**A**); 10 μm (**B–J**); 5 μm (**K**).

**Figure 10.**
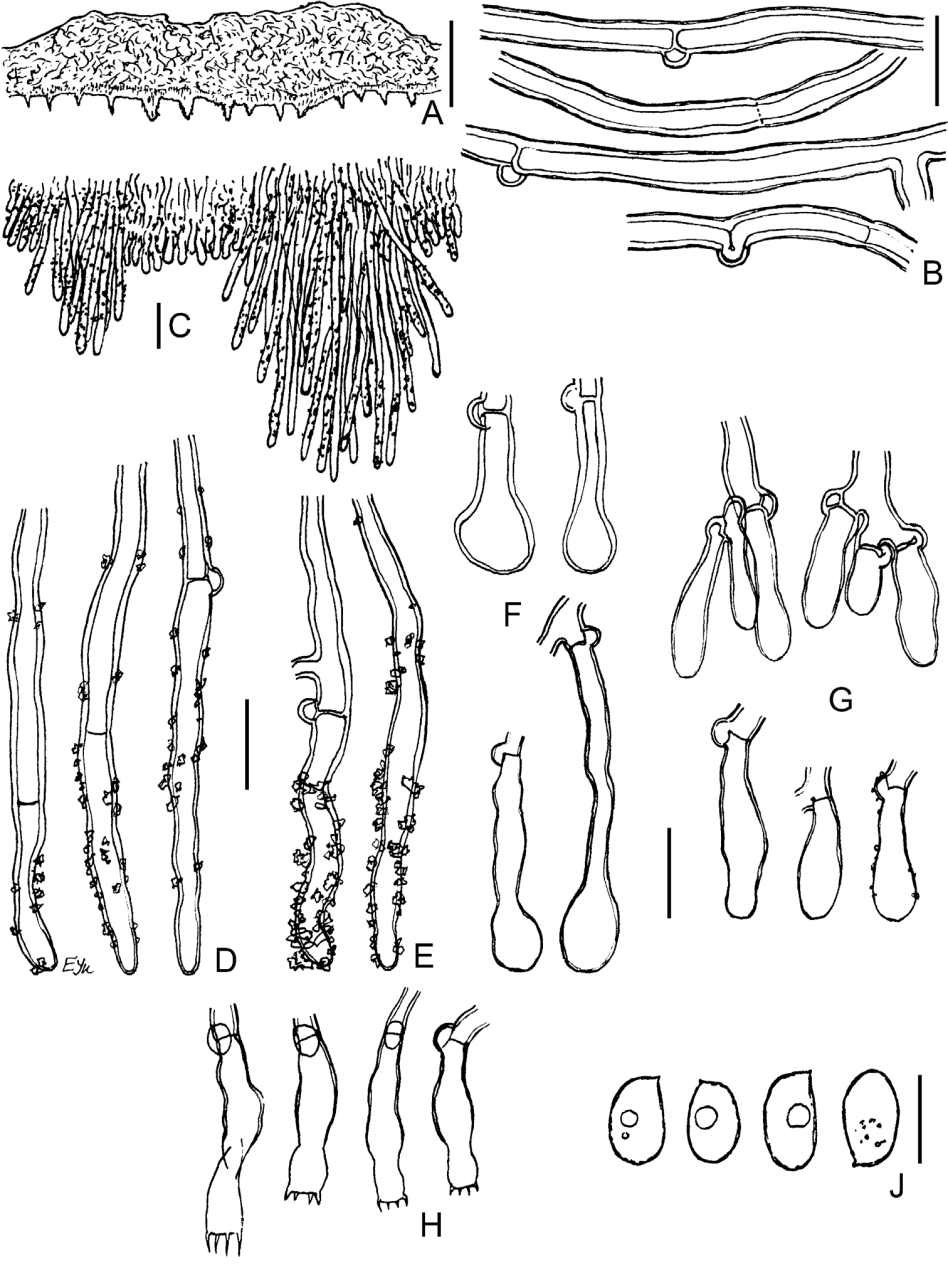
Micromorphology of *Xylodonlanatus* (CFMR: HHB-8925, holotype): **A** vertical section through basidiomata **B** subicular hyphae **C** vertical section through aculei and hymenium **D** projecting hyphae in 3% KOH**E** projecting hyphae in Mz**F** capitate cystidia **G** basidioles **H** basidia **J** basidiospores. Scale bars: 500 μm (**A**); 20 μm (**C**); 10 μm (**B, D–H**); 5 μm (**J**).

This species was considered conspecific with *Xylodonlanatus* from North America (Burdsall and Nakasone 1981, Wu 1990, Gorjón and Greslebin 2012), but we observed significant morphological differences. For example, in *X.vesiculosus*, the basidiomata have a denser, tough-membranaceous texture compared to the soft woolly basidiomata of *X.lanatus*. In addition, the aculei in *X.vesiculosus* are larger and the basidia are thin-walled in contrast to the smaller aculei and basally thick-walled basidia found in *X.lanatus* (compare Figs 9, 10). See Discussion for a key to *X.lanatus*, *X.vesiculosus* and allied taxa.

***Xylodonniemelaei* (Sheng H.Wu) Hjortstam & Ryvarden, Synopsis Fungorum 26: 28 (2009)**

≡ *Hyphodontianiemelaei* Sheng H. Wu, Acta Botanica Fennica 142:98 (1990).

***Xylodonrhizomorphus* (C.L.Zhao, B.K.Cui & Y.C.Dai) Riebesehl, Yurchenko & Langer, Mycological Progress 16(6): 649 (2017).**

≡ *Hyphodontiarhizomorpha* C.L.Zhao, B.K.Cui & Y.C.Dai, Cryptogamie, Mycologie 35(1):92 (2014).

#### Xylodon reticulatus

Taxon classificationFungiHymenochaetalesSchizoporaceae

(C.C.Chen & Sheng H.Wu) C.C.Chen & Sheng H.Wu, Mycoscience 59(5): 349 (2018).

≡ Hyphodontiareticulata C.C.Chen & Sheng H.Wu, Mycological Progress 16(5): 558 (2017).

##### Remarks.

Molecular and morphological analyses demonstrate that the three taxa listed above are very similar. The 11 samples of *X.niemelaei*, 3 of *X.rhizomorphus* and 3 of *X.reticulatus* formed a strongly supported clade (99 BS, 1 PP) in the ITS phylogram (Fig. 1). In addition, three samples representing two of the species are found in a strongly supported clade (100 BS, 1 PP) in the 28S tree (Fig. 2), differing in only one position in the associated alignment.

*Xylodonniemelaei* was described and illustrated in detail by Wu (1990) and Langer (1994). It is characterised by a poroid hymenophore, embedded and hymenial capitate cystidia, small, subulate or fusoid hymenial cystidia and encrusted hyphal ends mainly developed at the pore edges but sometimes also in other areas. At the morphological level, the bladder-like embedded cystidia and hyphal encrustations appear identical in *X.niemelaei* (Langer 1994), *X.rhizomorphus* (Zhao et al. 2014) and *X.reticulatus* (Chen et al. 2017). Spore size and spore quotient overlap in these three species. *Xylodonrhizomorphus* occurs in south-western China, whereas *X.reticulatus* occurs in Taiwan and Japan. *Xylodonniemelaei* is reported also from these three countries and furthermore from Réunion, Africa and South America (Langer 1994), but the last two reports require morphological and molecular confirmation.

##### Specimens examined.

*Xylodonniemelaei* – REUNION: Forêt Mare-Longue, on dead stump of angiosperm wood, leg. J. Riebesehl, M. Schröder, M.M. Striegel, 12 Mar 2015 (FR-0249846, dupl. as L1087 in KAS); on lumber, leg. J. Riebesehl, M. Schröder, M.M. Striegel, 12 Mar 2015 (FR-0249174, dupl. as L1077 in KAS); on brown-rotten wood, leg. E. Langer, G. Langer, E. Hennen, 21 Mar 1998 (KAS-GEL 4998); Forêt Notre-Dame de la Paix, on dead wood of *Monimiarotundifolia* Thouars, leg. E. Langer, 11 Mar 2013 (FR-0219860, dupl. as L0002 in KAS); on dead angiosperm wood, leg. J. Riebesehl, M. Schröder, M.M. Striegel, 10 Mar 2015 (FR-0249811, dupl. as L1031 in KAS); on white-rotten wood, leg. E. Langer, G. Langer, E. Hennen, 19 Mar 1998 (KAS-GEL 4904); Le Petit Tampon, on dead wood, leg. J. Riebesehl, M. Schröder, M.M. Striegel, 9 Mar 2015 (FR-0249225, dupl. as L1007 in KAS); Piton Mont Vert, on dead wood, leg. J. Riebesehl, M. Schröder, M.M. Striegel, 18 Mar 2015 (FR-0249178, dupl. as L1172 in KAS); Plaine des Fougères, on dead wood, leg. E. Langer, 12 Sep 2013 (FR-0249744, dupl. as L0698 in KAS); Sentier de Takamaka, on white-rotten wood, leg. J. Riebesehl, M. Schröder, M.M. Striegel, 26 Mar 2015 (FR-0249289, dupl. as L1269 in KAS).

#### Xylodon spathulatus

Taxon classificationFungiHymenochaetalesSchizoporaceae

(Schrad.) Kuntze, Revisio generum plantarum (Leipzig) 3(2):541 (1898)

≡ Hydnumspathulatum Schrad., Spicilegium Florae Germanicae: 178, t. 4:3 (1794).
= Hyphodontiabubalina Min Wang, Yuan Y.Chen & B.K.Cui, Phytotaxa 309(1):50 (2017).
≡ Xylodonbubalinus (Min Wang, Yuan Y.Chen & B.K.Cui) C.C.Chen & Sheng H.Wu, Mycoscience 59:349 (2018).
= Hyphodontiachinensis C.C.Chen & Sheng H.Wu, Mycological Progress 16(5): 554 (2017).
≡ Xylodonchinensis (C.C.Chen & Sheng H.Wu) C.C.Chen & Sheng H.Wu, Mycoscience 59: 349 (2018).

##### Remarks.

Based on both molecular data and morphology, we place the taxa *X.bubalinus* and *X.chinensis* in synonymy under *X.spathulatus*. In our phylogenetic analysis of ITS sequence data, the recently described *X.bubalinus* (4 collections) and *X.chinensis* (2 collections) from China form a well-supported clade with *X.spathulatus* (4 collections) from Europe (97 BS, 1 PP) that is sister to *X.apacheriensis* (Fig. 1). Within this clade are several subclades, with very low bootstrap support (<55), thus subspecies or varieties cannot be identified. The 28S rRNA gene analysis also supports conspecificity between *X.chinensis* and *X.spathulatus* (99 BS, 0.65 PP) (Fig. 2). *Xylodonspathulatus* has three main diagnostic features: prominent (1–2 mm tall) aculei of varied shape, numerous apically acute cystidia with 1–4 slight constrictions and capitate cystidia with a resinous cap. It is described and illustrated by Eriksson and Ryvarden (1976) and Langer (1994). Minor morphological variation amongst the three taxa was observed. For example, *X.chinensis* has ventricose cystidia, similar to those in *X.spathulatus*, but they are sometimes septate at the constrictions. Distinctly ventricose cystidia were not observed in *X.bubalinus*, which instead had hyphoid or subulate cystidioles (Wang and Chen 2017, Fig. 2f). Encrusted hyphal ends at apices of the aculei in *X.bubalinus* and *X.chinensis* are typical of those in *X.spathulatus*. Resinous caps enclosing capitate elements are often absent as in the case of *X.bubalinus*, *X.chinensis*, *X.spathulatus* KAS-GEL2690 (from Germany) and *X.spathulatus* MSK-F 12931 (from Russia). Spore shape and size are similar amongst the three taxa and the spore quotient 1.3–1.4(–1.5) overlaps (Eriksson and Ryvarden 1976, Wang and Chen 2017, Chen et al. 2017). A few spores in *X.chinensis* were up to 6 × 5 μm and may be due to better climatic conditions. The description of *X.spathulatus* is modified to include variable aculei from conical and subulate to distinctly spathuliform and the variable presence of cystidia with resinous caps, mucronate apices and a submoniliform type that are aseptate with more or less blunt apices. Thus *X.spathulatus* is a highly variable but distinctive species that is widely distributed from northern Europe (Eriksson and Ryvarden 1976) to southern China (Chen et al. 2017) and has a preference for old-growth forests (Dvořák et al. 2017). Reports of *X.spathulatus* from North and South America (Ginns and Lefebvre 1993, Hjortstam and Ryvarden 2007b) should be confirmed by molecular sequence data.

##### Specimens examined.

*Xylodonspathulatus* – CZECH REPUBLIC: Zofinsky National Park, on dead deciduous wood, leg. M.M. Striegel, 16 Sep 2015 (KAS-MMS 7224); GERMANY: Baden-Wurttemberg, Bad Waldsee, on dead wood of *Piceaabies* (L.) H.Karst., leg. E. Langer, G. Langer, 15 Oct 1992 (KAS-GEL 2690); SWEDEN: Gästrikland, Island Torrö, on dead wood of *Betula* sp., leg. K.H. Larsson, 29 Sep 1988 (GB KHL 7085, dupl. in KAS); RUSSIA: Udmurtia, near Izhevsk town, on *Sorbusaucuparia* L., leg. V.I. Kapitonov, 7 Aug 2012 (MSK-F 12931).

#### Xylodon cystidiatus

Taxon classificationFungiHymenochaetalesSchizoporaceae

(A.David & Rajchenb.) Riebesehl & Langer, Mycological Progress 16(6):645 (2017)

≡ Schizoporacystidiata A. David & Rajchenb., Mycotaxon 45:140 (1992).

##### Remarks.

**Figure 11.**
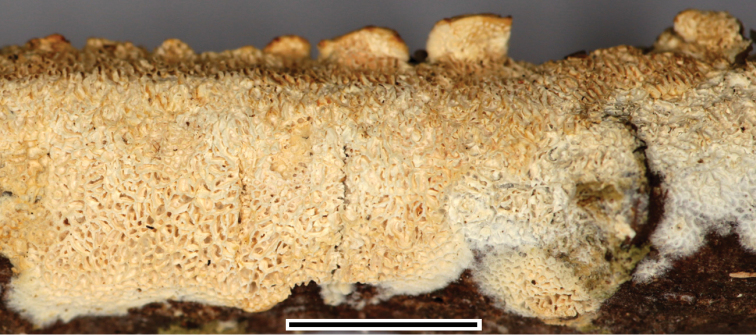
Basidioma of *Xylodoncystidiatus* (FR-0249200). Scale bar: 1 cm.

**Figure 12.**
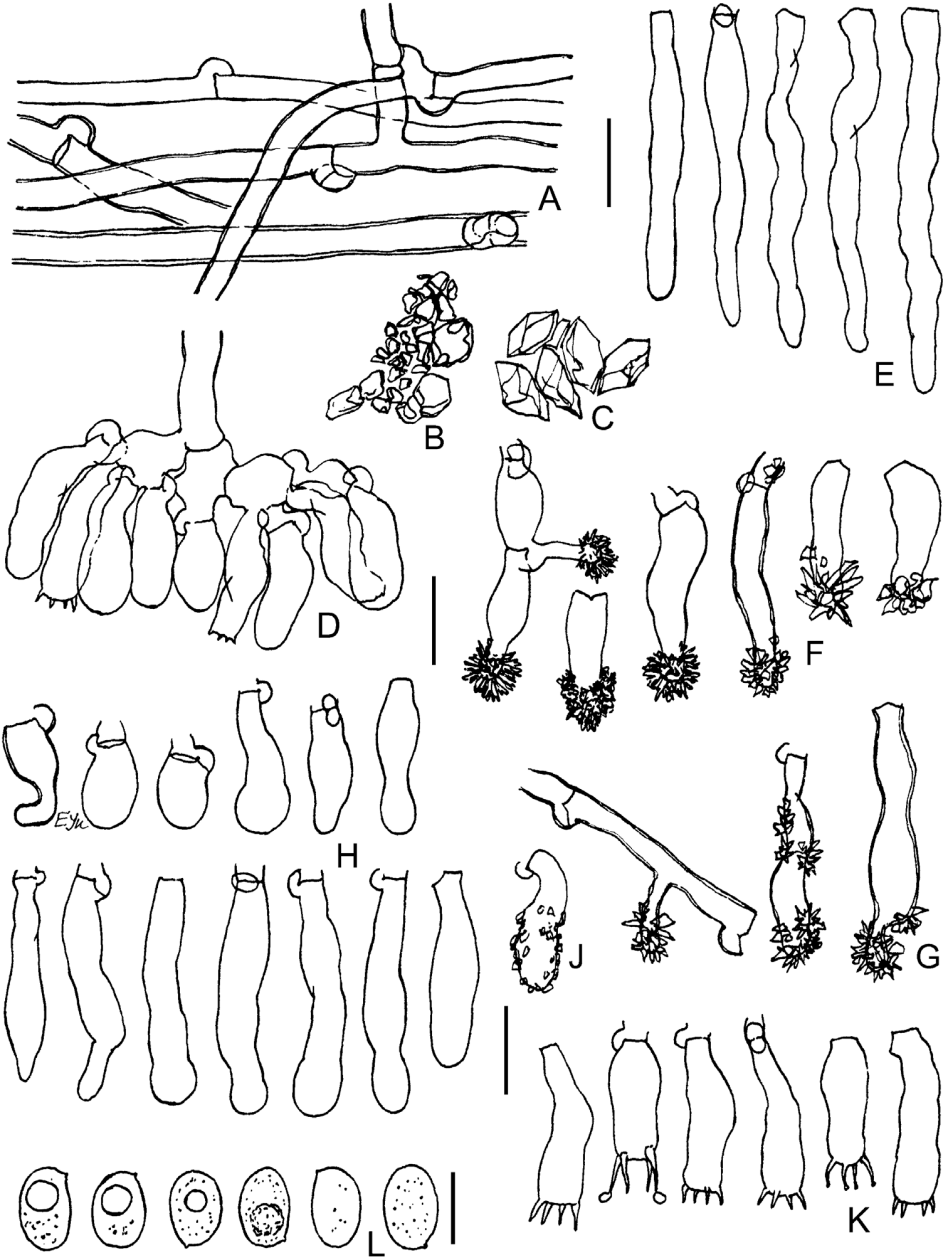
Micromorphology of *Xylodoncystidiatus* (FR-0249200): **A** subicular hyphae **B** crystals from dissepiment in 3% KOH**C** crystals from dissepiment in Mz**D** portion of hymenium and subhymenium **E** hyphal endings from dissepiment edges **F** encrusted cystidia in 3% KOH**G** encrusted cystidia in Mz**H** smooth basidioles and cystidioles **J** encrusted basidiole (in Mz) **K** basidia **L** basidiospores. Scale bars: 10 μm (**A–K**); 5 μm (**L**).

We undertook a thorough morphological analysis of the specimen FR-0249200 (Réunion, Plaine des Fougères, on fallen angiosperm twig, leg. E. Langer, 12 Sep 2013), because it provided the first sequences of *X.cystidiatus*. We are confident that FR-0249200 is *X.cystidiatus*, although we detected minor differences from the descriptions in David and Rajchenberg (1992) and Langer (1994). Some of the differences we noticed include: (1) the encrusted cystidia in FR-0249200 are mostly thin-walled with finer crystals; (2) the spores in our specimen were slightly broader 5–6 × 3.5–4.3 µm (L = 5.4 µm, W = 3.9 µm, Q = 1.4) than in published records 5–6 × 3–4 µm. Photographs of the basidioma (Fig. 11) and drawings of the microscopic features (Fig. 12) of FR-0249200 are provided for future identifications.

## Discussion

We recognise 77 species of *Xylodon* based on studies by Riebesehl and Langer (2017), Chen et al. (2017, 2018), Kan et al. (2017), Wang and Chen (2017) and results herein (Table 1). Our phylogenetic analyses included 122 ITS and 28S sequences representing 37 *Xylodon* species. The other 40 accepted species in *Xylodon* are based on morphological studies.

In the following discussion, we highlight some of the significant results.

### *Odontiopsis* is a synonym of *Xylodon*

The monotypic *Odontiopsis* Hjortstam & Ryvarden was described in 1980 based on *O.hyphodontina* from Tanzania. Hjortstam (1987) transferred *Hydnumambiguum* to *Odontiopsis* and placed *O.hyphodontina* in synonymy. Later, *Pteridomycessphaericosporus* was placed in synonymy with *O.ambigua* by Hjortstam (1991). Analyses of ITS and 28S sequences placed specimens originally identified as *O.ambigua* in the *Xylodon* lineage. Due to nomenclature rules to choose the earliest possible epithet to represent a taxon (see Art. 11.4 in Turland et al. 2018), the name for this taxon is *Xylodonhyphodontinus* and *Odontiopsis* is reduced to a synonym of *Xylodon*.

### *Palifer* is a synonym of *Xylodon*

Species in *Palifer* have apically encrusted cystidia that are characteristic of the genus and distinctly different from the lagenocystidia of *Hyphodontia* s.s. (Hjortstam and Ryvarden 2009, Riebesehl and Langer 2017). *Palifer* is defined primarily by morphology because there is only a single ITS sequence available. Phylogenetic studies place *P.verecundus* amongst the *Xylodon* species (Larsson et al. 2006, Fig. 1). The recently described *X.mollissimus* has cystidia that are similar to those of *Palifer* species and ITS sequence analyses place it in a clade with *Xylodon* sp. 1 (Kan et al. 2017, Fig. 1). Although not closely related, *X.mollissimus* and *P.verecundus* are embedded within *Xylodon* and demonstrate that the distinctive cystidia developed in *Palifer* is not a phylogenetically significant character. Thus, we reduce *Palifer* to a synonym of *Xylodon* and propose the following transfers:

#### Xylodon erikssonii

Taxon classificationFungiHymenochaetalesSchizoporaceae

(M.Galán & J.E.Wright) Riebesehl & Langer comb. nov.

MB827760

Grandinia erikssonii M.Galán & J.E.Wright, in Galán, Lopez & Wright, Darwiniana 32(1–4):251 (1993) (Basionym).
≡ Hyphodontiaerikssonii (M.Galán & J.E.Wright) Hjortstam & Ryvarden, Synopsis Fungorum 20: 63 (2005).
≡ Palifererikssonii (M.Galán & J.E.Wright) Riebesehl, Yurchenko & Langer, in Riebesehl & Langer, Mycological Progress 16(6): 646 (2017).

##### Typus.

ARGENTINA, Prov. Bonariae, Videla Dorna, on *Salixbabylonica* L., May 1972, Deschamps et al. (BAFC 31920 – holotype).

#### Xylodon gamundiae

Taxon classificationFungiHymenochaetalesSchizoporaceae

(Gresl. & Rajchenb.) Riebesehl & Langer comb. nov.

MB827761

Hyphodontia gamundiae Gresl. & Rajchenb., Mycologia 92(6):1159 (2000) (Basionym).
≡ Palifergamundiae (Gresl. & Rajchenb.) Hjortstam & Ryvarden, Synopsis Fungorum 22: 9 (2007).

##### Typus.

ARGENTINA, Tierra del Fuego, Dpto. Ushuaia, Estancia El Valdéz, on *Nothofaguspumilio* (Poepp. & Endl.) Krasser, 4–5 Mar 1996, A. Greslebin (BAFC 50036 – holotype).

#### Xylodon hjortstamii

Taxon classificationFungiHymenochaetalesSchizoporaceae

(Gresl. & Rajchenb.) Riebesehl & Langer comb. nov.

MB827762

Hyphodontia hjortstamii Gresl. & Rajchenb., Mycologia 92(6):1160 (2000) (Basionym).
≡ Paliferhjortstamii (Gresl. & Rajchenb.) Hjortstam & Ryvarden, Synopsis Fungorum 22: 9 (2007).

##### Typus.

ARGENTINA, Tierra del Fuego, Parque Nacional Tierra del Fuego, Río Pipo, on *Nothofagus* sp., 7 Nov 1998, A. Greslebin (BAFC 50037 – holotype).

#### Xylodon septocystidiatus

Taxon classificationFungiHymenochaetalesSchizoporaceae

(H.X.Xiong, Y.C.Dai & Sheng H.Wu) Riebesehl & Langer comb. nov.

MB827764

Hyphodontia septocystidiata H.X.Xiong, Y.C. Dai & Sheng H.Wu, Mycologia 102(4):918 (2010) (Basionym).
≡ Paliferseptocystidiatus (H.X.Xiong, Y.C.Dai & Sheng H.Wu) Riebesehl, Yurchenko & Langer, in Riebesehl & Langer, Mycological Progress 16(6): 649 (2017).

##### Typus.

TAIWAN, Taipei, Kungliao, on rotten angiosperm branch, 25 Nov 1990, Y.F. Lin (TNM Lin 90202 – holotype).

#### Xylodon verecundus

Taxon classificationFungiHymenochaetalesSchizoporaceae

(G.Cunn.) Yurchenko & Riebesehl comb. nov.

MB827765

Peniophora verecunda G.Cunn., Transactions and Proceedings of the Royal Society of New Zealand 83(2):262 (1955) (Basionym).
≡ Paliferverecundus (G.Cunn.) Stalpers & B.K.Buchanan, New Zealand Journal of Botany 29(3): 339 (1991).
≡ Hyphodontiaverecunda (G.Cunn.) Hjortstam & Ryvarden, Mycotaxon 64: 237 (1997).

##### Typus.

NEW ZEALAND, Auckland, Hauhangaroa Range, Taupo, on decayed decorticated wood of *Dacrydiumcupessinum* Sol., Mar 1953, J.M. Dingley (PDD 12513 – holotype).

### Notes

The three species *Paliferrickii* (Hjortstam & Ryvarden) Riebesehl, Yurchenko & Langer, *P.seychellensis* Dämmrich & Rödel and *P.wrightii* (Hjortstam & Ryvarden) Hjortstam & Ryvarden are today already accepted in other genera as *Xylodonrickii* (Hjortstam & Ryvarden) K.H. Larss. (Viner et al. 2018), *Sceptruluminflatum* (Burt) K.H. Larss. (Larsson 2014) and *Hyphodontiawrightii* Hjortstam & Ryvarden (Gorjón 2012).

### *Xylodonlanatus* and allied species

*Xylodonlanatus* was originally described by Burdsall and Nakasone (1981) based on collections from North America and New Zealand. A comparative morphological study of specimens, annotated as *X.lanatus* from Taiwan and North America, revealed that *X.lanatus* is a complex of morphologically similar species. The New Zealand specimen, *X.vesiculosus*, was discussed above and is considered to be a distinct species. The specimen *X.lanatus* (TUB-FO 40734) from Taiwan, depicted in Langer (1994), is *X.exilis*. The specimen of *X.lanatus* cited by Hjortstam and Ryvarden (1984) from Nepal is also *X.exilis*. In the protologue of *X.lanatus* (Burdsall and Nakasone 1981), the authors illustrated the paratype (HHB-6925 from Florida, U.S.A.) which is correctly identified as *X.pseudolanatus*. Hjortstam and Bononi (1987) reported *X.lanatus* from Brazil while noting the controversial taxonomic position of this species.

We accept *X.lanatus*, based on the type (CFMR HHB-8925; Figs 3f, 10) and paratype (CFMR HHB-4305), as a distinct species but with a restricted concept. We retain the same diagnostic features, noted in the protologue (basidiomata with well-developed woolly subiculum, terminal vesicular structures on subicular hyphae, poorly differentiated subhymenium, encrusted thick-walled hyphae in tooth apices and capitate cystidia) and add that walls of basidia and subhymenial hyphae directly under hymenial elements are slightly but distinctly thickened. The illustration of *Xylodonlanatus* from Taiwan, provided by Wu (1990), also shows basidia with walls thickened below, but hyphal pegs appear different from *X.lanatus* s.s. We have also determined that *X.echinatus* (Yurchenko et al. 2013) is the most morphologically similar species to *X.lanatus* s.s. A key to the taxa in the *X.lanatus* group is presented here.

**Table:** 

1	Basidioma between aculei 0.3–0.5 mm thick, woolly; subhymenial hyphae somewhat thick-walled directly under hymenium	**2**
–	Basidioma between aculei 0.05–0.15 mm thick, membranaceous or subceraceous; subhymenial hyphae thin-walled	**3**
2	Capitate cystidia present; basidia with slightly thickened walls in lower ½–2/3; spores 3–3.5 μm broad, Q = 1.8–2	*** X. lanatus ***
–	Capitate cystidia absent; basidia thin-walled; spores 3.5–4(–5) μm broad, Q = 1.6–1.9	*** X. echinatus ***
3	Subicular hyphae strongly thick-walled (up to 1.5 μm thick), often with narrow lumen; hymenophoral aculei 0.13–0.35 mm long, 4 per mm	*** X. vesiculosus ***
–	Subicular hyphae moderately thick-walled (up to 1–1.2 μm thick), with wide lumen; hymenophoral aculei 0.03–0.12 mm long, 8–14 per mm	**4**
4	Projecting hyphae in aculei strongly flexuous, thick-walled (up to 1–1.5 μm thick) in middle and lower part, provided with closely arranged simple and clamped septa, constricted at septa	*** X. exilis ***
–	Projecting hyphae in aculei slightly flexuous, slightly thick-walled, with remote septa, not constricted at septa	*** X. pseudolanatus ***

### *Xylodonniemelaei*, *X.reticulatus* and *X.rhizomorphus* are very closely related

Phylogenetic analyses of ITS sequences of 17 samples, including 8 new sequences of *X.niemelaei* and 28S sequences of three samples, demonstrate that the three taxa are very similar (Figs 1, 2). ITS sequences from holotypes of *X.reticulatus* and *X.rhizomorphus* were included in the analyses (Fig. 1). The ITS sequences were 98.2–99% similar amongst the taxa, differing at 6–11 sites. Minor morphological differences were noted amongst the taxa. We keep the taxa *X.niemelaei*, *X.reticulatus* and *X.rhizomorphus* as separate species following the results of phylogenetic studies of Chen et al. (2017) and Fernández-López et al. (2018a). The last work was published shortly before the completion of our study and therefore could not be considered further.

However, in our reconstruction, the phylogenetic distances between these taxa are very short, and comparable to those between the OTUs of *X.spathulatus*. Taking into account that these three taxa remain as monophyletic branches, we suppose that they can be subspecies or varieties of one species.

**Figure 13.**
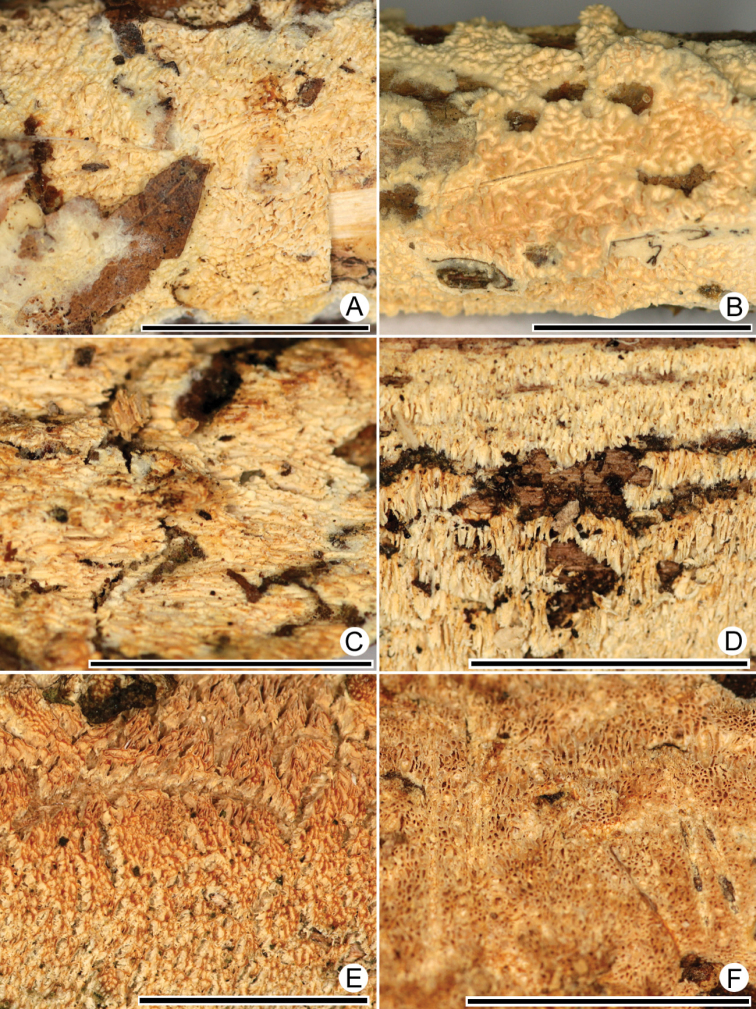
Hymenophores of *X.raduloides*. **A** KAS-JR 02, Germany **B** KAS-JR 03, Germany **C** KAS-JR 09, Germany **D** KAS-JR 26, Germany **E** KAS-JR 10, Germany **F** LR18813, Australia. Scale bars: 1 cm.

### *Xylodonbubalinus*, *X.chinensis* and *X.spathulatus* are conspecific

Phylogenetic analyses of ITS sequences of 10 samples, including sequences from holotypes of *Xylodonbubalinus* and *X.chinensis* and 28S sequences of *X.spathulatus* and holotype of *X.chinensis* show that the three taxa are conspecific (Figs 1, 2). Amongst the taxa, ITS sequences were 98.7–99.8% similar, differing at up to 8 sites. The hymenophore is quite variable in this group and the presence of the different types of cystidia is also variable. The correct name for this group is *X.spathulatus* with *X.bubalinus* and *X.chinensis* reduced to synonyms.

### The classification of *Xylodonaustralis* in *Xylodon* is confirmed

*Xylodonaustralis* is sequenced for the first time and shown in the 28S phylogenetic tree (Fig. 2) and its placement in *Xylodon* is confirmed. The sequenced specimen is from Argentina and was studied by Greslebin et al. (2000). They reported differences in spore morphology in specimens from Argentina, Australia and New Zealand. A molecular study may be able to resolve this species complex.

### The paratype material of *Xylodondimiticus* may be an independent species

Chen et al. (2017) showed that the holotype material of *Xylodondimiticus* (Jia J.Chen & L.W.Zhou) Riebesehl & Langer Dai 11686 is conspecific with *X.nongravis*. In addition, they also proposed that the paratype material Dai 15321 may be an independent species as shown in the phylograms. We support this view and included Dai 15321 in our phylograms as *Xylodon* sp. 1. The NCBI BLAST search of the ITS sequence of Dai 15321 shows an identity of 93% with the *X.dimiticus* holotype material Dai 11686 as well as with sequences of *X.nongravis*. Although this low similarity value indicates that Dai 15321 is a different species, a further study is needed to identify morphological differences.

### Additions to the distribution and morphology of *Xylodonserpentiformis*

A BLAST search of the newly generated *Xylodonserpentiformis*ITS sequences revealed that they are 99% identical to a sequence from South Korea identified as *Hyphodontia* sp. (KUC20121019-31, Jang et al. 2016). *Xylodonserpentiformis* is known from Taiwan and the Canary Islands (Langer 1994). Based on the small distance between Taiwan and South Korea and the similarities of the sequences, the distribution of *X.serpentiformis* is expanded to include South Korea. Langer (1994) cited a specimen of *X.serpentiformis* from the Canary Islands, but this material needs molecular confirmation. A distinctive feature, described for this species, was the presence of flexuous, thick-walled tubular tramacystidia in the aculei (Langer 1994). After our morphological analysis of the holotype of *Hyphodontiaserpentiformis* Langer (TUB-FO 40677) and three more specimens (TUB-FO 40675, TUB-FO 40985, TUB-FO 42688), we emend the diagnosis of *X.serpentiformis* as follows: aculei consisting mostly of flexuous, agglutinated projecting hyphae, hyphae slightly thickened or moderately thick-walled at base, then thinning toward apex and partly collapsing at maturity; spores broadly ellipsoid, ellipsoid, subovoid, sometimes narrowly ellipsoid.

### Sequences of *Xylodonraduloides* form two subclades in phylogenetic trees

The two subclades of *Xylodonraduloides* in the ITS phylogeny (Fig. 1) appear also in the 28S phylogram (Fig. 2), although with the inclusion of the sister species *X.subtropicus*. Both subclades include specimens from Germany and Australia. Micromorphological distinctions between the clades were not observed, but we noted that, in one of the clades (KAS-JR 02, 03, 09, 26), the hymenophore is pale cream to cream whereas, in the second clade (KAS-JR10, LR 18813), it is yellow-brownish (Fig. 13). Nevertheless, the morphological as well as sequence-based differences are not sufficient to recognise two separate species.

### Sequences of *Xylodonflaviporus* form two subclades in phylogenetic trees

In the ITS phylogram, two subclades are also present in the *Xylodonflaviporus* lineage (Fig. 1). Subclade 1 (FR-0249797, KAS-GEL 5047) comprises specimens from Réunion whereas subclade 2 (FCUG 1053, KAS-GEL 3462) comprises specimens from Romania and Taiwan. All other ITS sequences of *X.flaviporus* from the NCBI GenBank are from the northern hemisphere (Romania, South Korea, Taiwan, Turkey and USA) and clustered together in subclade 2 (data in Fig. 1 are reduced to specimens from Romania and Taiwan). We did not find micromorphological differences between specimens of the two subclades and the variation in the ITS sequences is too small to merit recognition of two different species.

### *Xylodonramicida* and *X.quercinus*

*Xylodonramicida* and *X.quercinus* are morphologically similar (Ariyawansa et al. 2015), exhibiting slight differences in spore width and shape and different substrate preferences. Their ITS sequences are 98.8–99% similar, differing at just 6–7 positions (*X.quercinus*: Miettinen 15050 and *X.ramicida*: Spirin 7664). Taking into account the similarity values of other *Xylodon* species (*X.spathulatus* 98.7–99%, *X niemelaei* 98.2–99%) and small morphological differences between *X.ramicida* and *X.quercinus*, we believe that *X.ramicida* is a well-defined subspecies within *X.quercinus*. More sequences from both taxa are required, however, before the taxonomic status of *X.ramicida* can be clarified.

### The taxonomic status of *Xylodondetriticus*

In this study, we accept *Hyphodontiadetritica* (Bourdot) J. Erikss. in *Xylodon* as *X.detriticus*. This combination was introduced by Ţura et al. (2011), recognised as invalid in MycoBank (Art. 36.1a and b, Melbourne Code) and supported in the work by Rosenthal et al. (2017). The first sequenced specimen of this species was GB Nilsson 990902 (Larsson 2007). We have studied this specimen and it is identical to the concept of *Hypochniciumdetriticum* (Bourdot) J. Erikss. & Ryvarden (Eriksson and Ryvarden 1976). The alignment between GB Nilsson 990902 and *X.detriticus* UC2023108 (Rosenthal et al. 2017) in ITS2 (ITS1 is unavailable for the previous specimen) showed nearly 100% similarity. We discovered that the ITS2 sequences between *Lagarobasidiumdetriticum* MA-Fungi 5758 (Dueñas et al. 2009) and GB Nilsson 990902 were 62% identical. Consequently, the taxonomic identity of *Lagarobasidiumdetriticum* MA-Fungi 5758 needs to be investigated. Viner et al. (2018) came to the same conclusion, but we could not integrate their further results, because this study was already finished when the work of Viner et al. was published.

## Conclusion

The usefulness of ITS sequences alone in defining and identifying species in *Xylodon* is approaching its limits. Further studies in *Xylodon* will require sequences from additional genetic markers with more variation. Fernández-López et al. (2018b) published the first phylogenetic tree for *Xylodon* with rpb2 sequences, but it contains only sequences of six different species. Nevertheless, the topology is very similar to our ITS and 28S trees.

Morphological features for defining species in *Xylodon* is also limited. Species, such as *X.spathulatus* with its variability in aculei morphology and in cystidia occurrence and shape, present challenges for identification. In other cases such as *X.hyphodontinus*, ITS sequence differences are significant whereas morphological differences are elusive.

## Supplementary Material

XML Treatment for Xylodon exilis

XML Treatment for Xylodon filicinus

XML Treatment for Xylodon follis

XML Treatment for Xylodon pseudolanatus

XML Treatment for Xylodon hyphodontinus

XML Treatment for Xylodon vesiculosus

XML Treatment for Xylodon reticulatus

XML Treatment for Xylodon spathulatus

XML Treatment for Xylodon cystidiatus

XML Treatment for Xylodon erikssonii

XML Treatment for Xylodon gamundiae

XML Treatment for Xylodon hjortstamii

XML Treatment for Xylodon septocystidiatus

XML Treatment for Xylodon verecundus
